# DNA-Dye-Conjugates: Conformations and Spectra of Fluorescence Probes

**DOI:** 10.1371/journal.pone.0160229

**Published:** 2016-07-28

**Authors:** Frank R. Beierlein, Miguel Paradas Palomo, Dmitry I. Sharapa, Oleksii Zozulia, Andriy Mokhir, Timothy Clark

**Affiliations:** 1 Computer-Chemistry-Center and Interdisciplinary Center for Molecular Materials, Department of Chemistry and Pharmacy, Friedrich-Alexander-Universität Erlangen-Nürnberg, Erlangen, Germany; 2 Cluster of Excellence Engineering of Advanced Materials, Friedrich-Alexander-Universität Erlangen-Nürnberg, Erlangen, Germany; 3 Organic Chemistry II, Department of Chemistry and Pharmacy, Friedrich-Alexander-Universität Erlangen-Nürnberg, Erlangen, Germany; Helsingin Yliopisto, FINLAND

## Abstract

Extensive molecular-dynamics (MD) simulations have been used to investigate DNA-dye and DNA-photosensitizer conjugates, which act as reactants in templated reactions leading to the generation of fluorescent products in the presence of specific desoxyribonucleic acid sequences (targets). Such reactions are potentially suitable for detecting target nucleic acids in live cells by fluorescence microscopy or flow cytometry. The simulations show how the attached dyes/photosensitizers influence DNA structure and reveal the relative orientations of the chromophores with respect to each other. Our results will help to optimize the reactants for the templated reactions, especially length and structure of the spacers used to link reporter dyes or photosensitizers to the oligonucleotides responsible for target recognition. Furthermore, we demonstrate that the structural ensembles obtained from the simulations can be used to calculate steady-state UV-vis absorption and emission spectra. We also show how important quantities describing the quenching of the reporter dye *via* fluorescence resonance energy transfer (FRET) can be calculated from the simulation data, and we compare these for different relative chromophore geometries.

## Introduction

**Fig 1:**
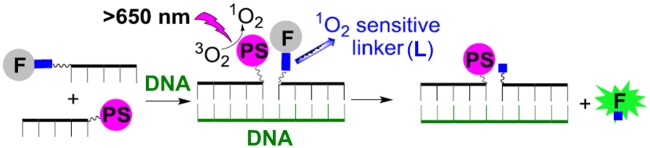
Detecting specific nucleic acid sequences in a templated reacion. **F**: 5-carboxyfluorescein, **PS**: photosensitizer (pyropheophorbide-a, PPa), **L**: linker cleavable by ^1^O_2_. [10, 25–27]

**Fig 2:**
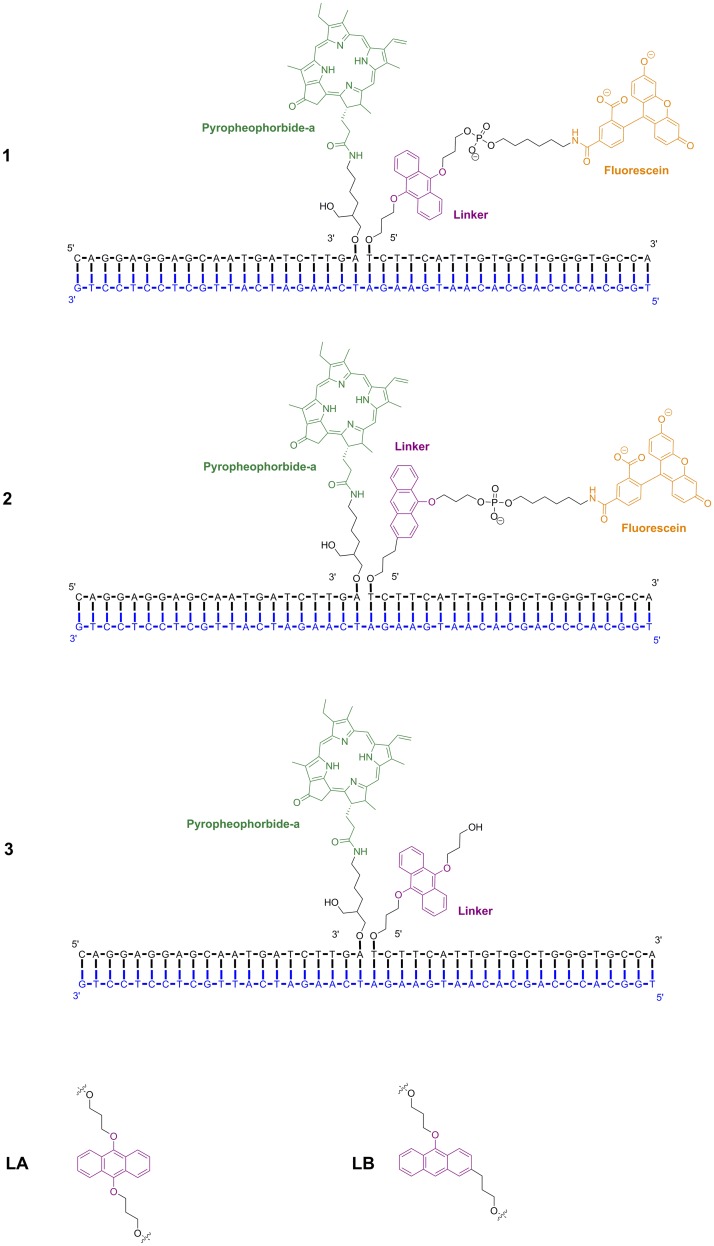
1–3: Structures of DNA-dye conjugates (green: PPa, orange: fluorescein) with linkers (magenta) cleavable by ^1^O_2_. **LA, LB**: linkers: 1,9-dialkoxyanthracene (left) and 9-alkoxyanthracene derivatives (right).

Oligonucleotides with covalently bonded fluorescence dyes and photosensitizers can be used as probes to identify and localize specific DNA- or RNA-nucleotide sequences *in vitro* and in live cells. [1–9] Examples of such systems are shown in Figs 1 and 2. [10, 11] Here, a photosensitizer (PS, e.g. pyropheophorbide-a, PPa) is conjugated to an oligonucleotide fragment comprising one half of the sequence complementary with the sequence of interest (the “target” or “template”; a DNA sequence representing a fragment of β-actin mRNA is frequently used as test target sequence) and a fluorescence dye (e.g. 5-carboxyfluorescein) is connected *via* an anthracene linker to the other. Upon addition of the complementary DNA or RNA strand (the template), a hybrid double-stranded oligonucleotide is formed, in which fluorescein fluorescence is quenched. Plausible quenching mechanisms are fluorescence resonance energy transfer (FRET, Förster energy transfer) [12, 13] to the photosensitizer and contact quenching, although a drop of fluorescence intensity is also caused by the mere presence of DNA or DNA plus linker. Several designs of the red-light-controlled, templated reaction have been constructed, some with a second dye as internal standard or additional fluorophore. [10, 11] Upon exposure of the complete system to red light, ^1^O_2_ is generated on the PS, which reacts with the linker thereby leading to its cleavage and fluorophore release (Fig 1). These reactions can be used to detect specific nucleic-acid sequences, even in complex mixtures such as cell lysates and live cells, or to monitor localization of the nucleic-acid template by, e.g., fluorescence microscopy or flow cytometry. [10, 11]

Modern “super-resolution” fluorescence imaging methods (e.g. stochastic (direct) optical reconstruction microscopy ((d)STORM), photoactivation-localization microscopy (PALM) and similar approaches) depend on the availability of target-specific, photoactivatable fluorescence probes. [14–24] Stochastic and light-controlled activation of a subset of fluorescence reporters, single-molecule imaging and image reconstruction are essential for many of these methods. [11] Transformations of dyes in photochemical, nucleic acid controlled reactions can potentially be used in single-molecule (SM)-microscopic imaging of natural nucleic acids as previously demonstrated for a model system. [11]

In the templated reactions studied here the cleavable, ^1^O_2_-sensitive linkers are 1,9-dialkoxyanthracenyl or 9-alkoxyanthracenyl fragments (Fig 2, **LA** and **LB**). [28, 29] The latter one has been shown to be the more effective because no external reducing agent is required to cleave the intermediate endoperoxide formed by cycloaddition of ^1^O_2_. [29]

**Fig 3:**
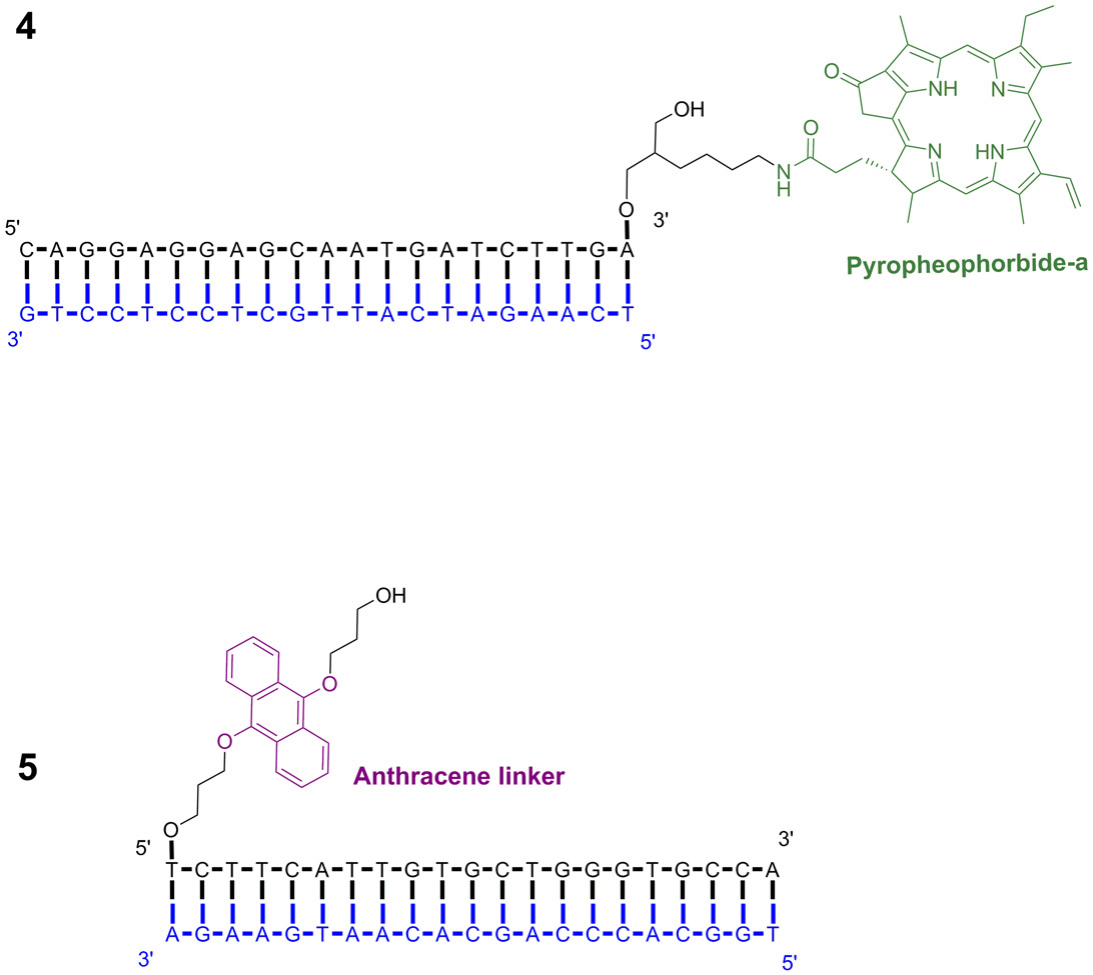
DNA-dye conjugates with terminally attached dyes.

We now report extensive molecular-dynamics (MD) simulations of a series of DNA-dye-conjugate systems. These systems are shown in Figs 2 and 3. In compounds **1** and **2**, pyropheophorbide-a (PPa) is attached as a photosensitizer to a DNA fragment complementary to β-actin-mRNA, whereas 5-carboxyfluorescein is conjugated to another DNA oligomer *via* a ^1^O_2_ –sensitive anthracene linker. We have also simulated a system lacking fluorescein (**3**).

The DNA-dye conjugates shown in Fig 2 resemble “complete” nucleic-acid detection systems comprising two probes (short single strands with attached PPa and dye/linker, respectively) and a complementary strand of the target sequence. In order to obtain closer insight into possible interactions of the chromophores when attached terminally at short oligonucleotides, we investigated control systems such as those shown in Fig 3. In the first control, one PPa is attached to the shorter DNA duplex (mimicking a catalytic part of the complete system, **4**) and in the second the anthracene linker is attached to the short DNA duplex (mimicking a substrate part of the complete system, **5**). The dye in these systems can interact in different ways with DNA, e.g., by π-π-stacking with the terminal base pair, intercalation between the bases, interaction with the phosphate backbone or contacts in the major or minor grooves. Additionally, the ring system of the chromophore can be inserted between the strands if the terminal base pairs are opened (“fraying”). These interactions, which affect the melting behavior, can be investigated by MD simulations. In order to determine the influence of PPa in **4**, we have also simulated a DNA fragment with identical sequence, but without PPa (**4-DNA**).

Preliminary experimental results indicate that FRET or contact quenching occurs between fluorescein and PPa (Fig 2, systems **1** and **2**). [30] Quenching of the photosensitizer (PPa) is not desired, as this would inhibit ^1^O_2_ production.

Because of its strong distance dependence, FRET [12, 13] is frequently used to examine molecular distances in biological macromolecules (“spectroscopic ruler”), [31, 32] and is an important technique for observing molecular interactions and conformational changes with high spatial and time resolutions. We have previously developed a method that combines classical MD simulations with quantum mechanical/molecular mechanical (QM/MM) [33, 34] calculations to simulate fluorescence spectra with and without FRET quenching. [35, 36] This approach was used to shed light on the important question of how conformational ensembles determine spectroscopic features, and it was shown that the common isotropic assumption made in many FRET studies must be corrected using simulation data. Calculated spectra are generally of high value as they provide a means to test different hypotheses in cases where experiments alone cannot explain the spectra satisfactorily. In addition to distance measurements, FRET can also be used in the sense of a binary readout, i.e., to investigate whether two chromophores are geometrically close to each other, or not. In that sense, FRET is frequently used in molecular diagnostics, e.g. in nucleic-acid detection.

Several computational studies of dyes attached to oligonucleotides have been published, [37–39] some of them investigating FRET. Donor-acceptor geometries (and, therefore mean values of the squared orientation factors) have been shown to depend on the attachment point (terminal 5’, terminal 3’, or nonterminal), the nature of the terminal base pair and the dye or linker. Molecular modelling helps to understand specific dye-DNA (or dye-RNA) interactions, e.g. stacking of the dye on the ends, [37] intercalation between base pairs, or interaction with the backbone.

Fluorescence labels can exhibit conformational flexibility, depending on the structure of the linkers or spacers by which they are attached to the biomolecule. This property minimizes the influence of the label side-chain on the biomolecule conformation, but it complicates the accurate prediction of inter-label distances. Simulation methods are therefore required that account for linker flexibility and enable reliable modelling of the fluorophore probe positions. [40]

Another important field of ongoing research is the investigation of fraying of the terminal base pairs in MD simulations of solvated DNA and RNA oligomers, which, other than in nature, are not stabilized by, e.g., histone proteins. Simulations on a microsecond time scale showed frequent disruption of the terminal base pair, exposure of the bases to solvent (“fraying”), and formation of non-canonical structures. [41] While some features of fraying are consistent with the available experimental data, others point toward potential problems with the force-field description of DNA and RNA molecules. None of the force fields tested provide a completely satisfactory description of the terminal regions, indicating that further improvement is needed to achieve realistic modelling of fraying in DNA and RNA molecules. [41] Also, “dangling ends”, i.e., unpaired, overhanging nucleotides [42] or terminally attached dyes, [37] are the subject of intensive research as they can have a pronounced effect on duplex stability.

In the current work, we present simulations of DNA-conjugates, i.e., flexible, complex systems in which a delicate balance of bonding and nonbonding interactions determines structure and properties. Our simulations were conducted in close collaboration with an experimental group and we compare our simulation results directly with the spectra and measurements obtained experimentally. A close interaction between experiment and theory is essential to understand the experimental data (spectra, structures, fluorescence-quenching mechanisms) fully. We describe a simulation protocol that can be used to optimize the experimental detection systems further. The features to be optimized include the linkers, spacers and reporter dyes or photosensitizers linked to the oligonucleotides responsible for target recognition, and their ideal relative orientations, i.e. the optimal number of base pairs between the dyes and the optimal spacer length. Our classical MD-simulations provide information about the dyes’ influence on DNA structure and reveal the relative orientations of the chromophores with respect to each other. The interactions of the dyes or linkers with the oligonucleotides to which they are attached are investigated in detail, and we analyze their influence on DNA/RNA properties. We also show how important quantities that describe FRET quenching can be calculated from the simulation data using a QM/MM approach, and we discuss these for different relative chromophore geometries. One important benefit of calculating FRET data from simulations is that we are able to obtain accurate values for the orientation factor and FRET efficiency even for subsets of the conformational ensemble. [35] While the current work focuses on steady-state FRET spectroscopy, we also intend to develop a detailed model of fluorescence kinetics.

## Computational Details

We have parameterized the force fields for the series of systems shown in Figs 2 and 3. While the DNA part of the systems is described by the Barcelona extension to the Amber [43] ff94/ff99 [44, 45] force field (ff99-bsc0), [46] the dyes, linkers and spacers that are covalently bonded to 5’ and 3’ ends required special attention. Charges were derived using a restrained electrostatic potential fit (RESP) procedure [44, 47–49] based on *ab initio* calculations (HF/6-31G*//B3LYP/6-31G*) [50–53] with Gaussian 09 [54] (optimizations were performed in polarizable continuum model (PCM) water); [55] in agreement with the Amber force fields. [44, 47, 48] All other parameters were derived from the GAFF force field and the literature. [56–58] Because the appropriate force fields are more reliable, initial simulations were carried out for DNA-based systems.

Classical molecular-dynamics simulations and analyses were performed using the AMBER 14 [43] software with AmberTools 14 and 15, following a protocol established previously. [59, 60] The DNA-dye conjugates were constructed using Materials Studio [61] and NAB from the AmberTools suite [43] and initially minimized with implicit solvent (GB/SA) for 100 steepest descent and 400 conjugate gradient steps. They were then solvated in truncated octahedral boxes of SPCE [62] water molecules, so that the simulation cells exceeded the solutes by 15 Å. Sodium ions were added to neutralize the systems, and additional NaCl was added to achieve a Na^+^ concentration of 150 mM. Joung/Cheatham parameters were used for the ions. [63] Typical systems sizes were up to 272,952 atoms (89,894 water molecules, system **2**).

Five thousand steps of geometry optimization were performed (500 steps of steepest decent and 4,500 steps of conjugate gradient) using weak restraints (50 kcal mol^−1^ Å^−2^) on the dye, linker and DNA atoms, followed by 5,000 steps without restraints. The optimized structures were used as input for Langevin dynamics simulations at different temperatures (typically 310 K, 37°C, and higher temperatures), using a 2 fs time step and a collision frequency of 2 ps^−1^. Bonds involving hydrogen were constrained using SHAKE. [64] The distance cutoff for all nonbonding interactions was set to 10 Å. Long-range electrostatics were described by the particle-mesh Ewald method. [65, 66] For van der Waals interactions beyond those included in the direct sum, a continuum model correction for energy and pressure was used, as implemented in Amber 14. System heat-up was performed during a 500 ps constant volume (NVT) simulation with weak restraints (10 kcal mol^−1^ Å^−2^) on the dye, linker and DNA atoms. The systems were then simulated at constant pressure (NPT) (1 bar, weak pressure coupling, isotropic position scaling, pressure relaxation time 2 ps) without any restraints. Systems **1** and **2** were simulated for 230.5 ns, detailed analyses were performed after the 0.5 ns NVT heat-up phase. System **3** was simulated at 310 K for 100.5 ns and system **4** for 270.5 ns at 310K. Additionally, a simulation of 220.5 ns at 363 K (90°C) was performed. System **4** without PPa (the pure DNA sequence, **4-DNA**) was simulated for 270.5 ns at 310 K and system **5** until 220.5 ns (at 310 K). Geometries (snapshots) were saved every 10 ps.

Standard MD analyses were performed using Cpptraj from the AmberTools suite [67] (RMSD, RMSF, analysis of interactions, distance measurements, hydrogen bond analyses). Special attention was paid to:

- possible fraying of terminal base pairs,
- intercalation of the dyes between neighboring, Watson-Crick paired base pairs (π-π-stacking),
- stacking of the dyes on terminal bases or dangling in solution,
- interaction with phosphates,
- other contacts in the major or minor groove,
- insertion between terminal base pairs that are not paired (i.e., frayed—in this case it is also possible that the dye is π-π-stacked with a base).

The overall structure and dynamics, like DNA bending, were also analyzed.

As we intend to investigate FRET and alternative quenching mechanisms that occur in the systems presented here in a more detailed subsequent study, we have adapted our approach to calculate absorption and emission spectra [35, 36] and have calculated steady-state spectra and transition dipoles (which are important for FRET) for the PPa and fluorescein chromophores in system **1**.

Different clustering algorithms to discriminate typical chromophore geometries to be used for the QM/MM simulations were compared. Here, RMSD-based clustering (as implemented in Cpptraj) [67] was compared to an approach in which the side chain dihedrals are analyzed as a Markov chain (DASH). [68] Initial tests showed that clustering (hierarchical agglomerative, average-linkage) according to RMSD of PPa, anthracene and fluorescein ring heavy atoms, calculated vs. the minimized starting structure, after fitting the whole trajectory on the DNA backbone atoms of the minimized starting structure, showed the best results. We performed QM/MM single-point calculations on all structures in all geometrical clusters obtained from the cluster analysis. As a reference, we selected a cluster with the chromophores exposed to the solvent (424 structures) and separated from each other, which most closely resembles the dyes fluorescein and PPa in solution.

The method we use here to calculate spectra was established in our previous work on protein fluorescence, [35, 36] in which we investigated the tetracycline-repressor (TetR)/tetracycline complex as an example of a protein with FRET quenching of a tryptophan (Trp) chromophore. A classical molecular-dynamics simulation gives “hot” geometries of the complex, which are the basis for combined quantum mechanical/molecular mechanical (QM/MM) [33, 34] single-point configuration-interaction (CI)-calculations using semiempirical MO-theory. The semiempirical CI-calculations provide all the variables required to calculate steady-state absorption and fluorescence spectra and to develop a detailed model of time-resolved fluorescence emission and fluorophore quenching by FRET. [35, 36] A detailed description of the formalism used is given in the supporting information of our previous paper, [35] so that we will only outline the major assumptions here. Vertical absorption and emission wavelengths are calculated. For the simulation of steady-state and time-resolved fluorescence transitions, we calculate the Einstein coefficient *A*_*nm*_ of spontaneous emission (which represents the rate constant *k*_*r*_ for the exclusive deactivation of the first excited singlet state S_1_ by fluorescence) for each snapshot from the transition moment and the wavelength of the transition. Steady-state fluorescence profiles of the entire ensemble (the whole MD trajectory or a subset) are obtained as follows: The Einstein coefficients *A*_*nm*_ are binned in wavelength intervals, overlaid with Gaussian functions (on the energy scale, FWHM = 0.04 eV), and the sums of the Gaussians are plotted *vs*. the emission wavelength. For the systems presented in the current work, we intend to calculate time-resolved fluorescence decays and a detailed model for FRET in a subsequent study. Absorption spectra are generated analogously to the steady-state fluorescence profiles by overlaying the binned oscillator strengths with Gaussians, and plotting the sums of these *vs*. the wavelength. All transitions S_0_ → S_i_ in all snapshots are considered. Wavelength data are calculated for the vertical transition between S_0_ and the target states. This approach neglects Franck-Condon effects completely and therefore gives narrower profiles than the peaks observed in the experimental spectra. The quantum mechanical/molecular mechanical (QM/MM) single point configuration interaction (CI)-calculations were performed for all MD snapshots from the previously selected cluster using the semiempirical program package VAMP [69] and the AM1 Hamiltonian. [70] The relevant chromophores were embedded in the environment and the solvent using a quantum mechanics/molecular mechanics (QM/MM)-CI-approach in which the environment is represented by a classical force field. The polarization of the QM wavefunction by the point charges in the MM environment is taken into account *via* an additional one-electron term in the Fock matrix. [71] For the preparation of the QM/MM inputs, the spacer/linker chain was cut using a modified link atom approach, as described previously. [35] The cutting schemes for the fluorescein and PPa chromophores are shown in Figures Y and Z in S1 File. Note that the QM/MM formalism leads to a situation in which the chromophores are embedded in an MM environment and the resulting spectra resemble those of the chromophore alone (in a point charge environment) and not those of the complete system with DNA. The QM/MM results (with explicit solvent) were also compared to those obtained using a continuum representation of the solvent, e.g. the self-consistent reaction field (SCRF)-technique (not shown). [72, 73]

Quantities that describe FRET were calculated as described previously. [35] The transition dipole describing emission from the fluorescein S_1_ state was used together with use the most intense vertical PPa absorption that occurs at longer wavelength than the donor emission together with an energy-difference cutoff, which serves as an implicit correction for errors in the calculated excitation energies and for the neglect of excited-state relaxation. From these dipoles, we calculated the orientation factor *κ*^*2*^, which describes the relative orientation of the donor and the acceptor transition dipoles. [32]

$$\kappa^{2}={\left(\text{cos}\Theta_{T}-3\text{cos}\Theta_{D}\text{cos}\Theta_{A}\right)}^{2}$$ (1)

*θ*_*T*_ is the angle between the emission transition dipole of the donor and the absorption transition dipole of the acceptor, *θ*_*D*_ and *θ*_*A*_ are the angles between these dipoles and the line connecting donor and acceptor. Depending on the relative orientation of donor and acceptor, *κ*^*2*^ can range from 0 (orthogonal dipoles) to 4 (collinear and parallel dipoles) and *κ*^*2*^ = 1 for parallel dipoles. If donor and acceptor rotate freely, the mean value of *κ*^*2*^ is 2/3, a value assumed in many studies. [32] This value is appropriate for solution studies of small molecules but not for molecules bound to proteins or nucleic acids, which can have very anisotropic orientational distributions.

We then calculated a relative FRET rate constant *k*_*T*,*rel*_. from the orientation factor and the donor-acceptor distance *r* for each MD snapshot.

$$k_{T,rel.}=\;\kappa^{2}\cdot \frac{1}{r^{6}}$$ (2)

A normalized overlap integral *J* was calculated from the experimental emission of carboxyfluorescein and the absorption of PPa (the experimental spectra were recorded for systems with the chromophores attached to DNA-21-mers, i.e. systems like **4** and a corresponding system with fluorescein (“the right half of system **1**”), using the software a|e 2.2 (1.044·10^15^ nm^4^/(M·cm)). [74] Together with the experimental value for the fluorescence quantum yield of the FRET donor *Q*_*D*_ (0.099), which was derived using the experimental emission spectrum described above together with the experimental fluorescence quantum yield of fluorescein, [75] we calculated a value for the Förster radius *R*_*0*_ for each snapshot using the computed orientation factor *κ*^*2*^ (Eq 1) of each snapshot: [32]

$$R_{0}=0.211{\left[\kappa^{2}n^{-4}Q_{D}J(\lambda )\right]}^{1/6}$$ (3)

$$R_{0}^{6}=8.79\;\cdot 10^{-5}[\kappa^{2}n^{-4}Q_{D}J(\lambda )]$$ (4)

If *J(λ)* is given in nm^4^/(M·cm), then *R*_*0*_ is obtained in Å. [32] Together with the distance *r* between the geometric centers of the chromophores (xanthene ring atoms in fluorescein and ring atoms in PPa), we can easily calculate a FRET efficiency for each snapshot: [32]

$$E=\frac{R_{0}^{6}}{R_{0}^{6}+r^{6}}$$ (5)

These relative FRET rate constants and efficiencies are sufficient for the aim of the current paper, where we compare different relative chromophore geometries with respect to their suitability for FRET quenching. For future studies, the relative FRET rate constant of each snapshot can be multiplied with a factor in order to obtain a rate constant distribution with a mean *k*_*T*_ value that follows from experimental investigations. These can be used together with data for alternative, nonradiative quenching pathways *(k*_*nr*_*)* and the Einstein coefficient of each snapshot *(k*_*r*_*)* to obtain a complete model of fluorescence kinetics, as in our previous work on protein fluorescence. [35]

## Results and Discussion

We first analyze and discuss the results of the classical MD simulations of all systems. We then use the conformational ensemble of system **1** to calculate steady-state absorption and emission spectra, and to calculate FRET between the chromophores in this system.

### Classical MD Simulations

**Fig 4:**
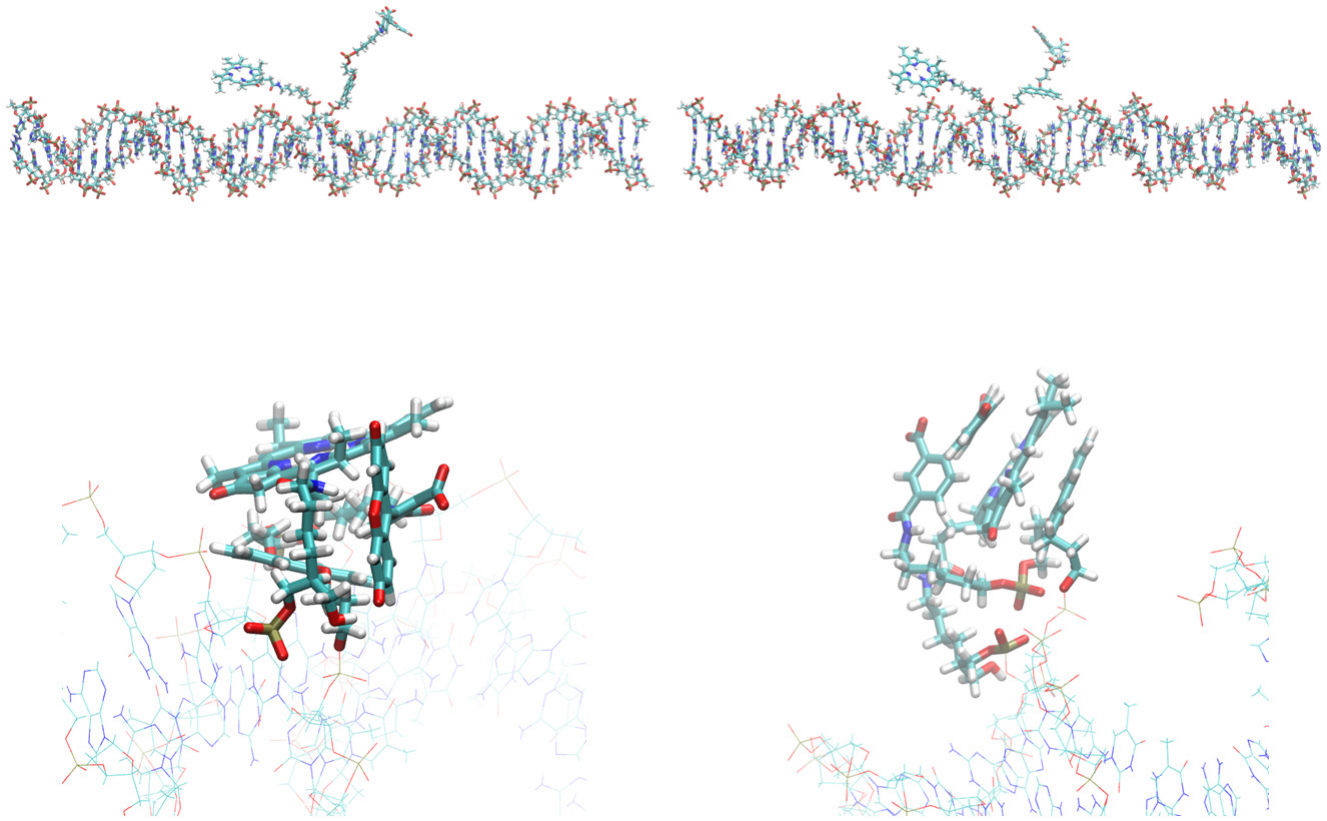
Structures of systems 1 (left) and 2 (right) at 0.51 ns (after equilibration with harmonic restraints, above) and at 230.5 ns (below, dyes and linkers shown as sticks, DNA as lines). Ions and water were omitted for clarity. Figures were created using VMD. [76]

To assess the influence of the two different linkers **LA** and **LB** on the relative orientations of the chromophores PPa and fluorescein, we first analyzed the conformational ensembles of systems **1** and **2**. In the starting structures of both systems (Fig 4), the chromophores dangled in the solvent and were separated by 32.7–39.0 and 34.7–40.9 Å for systems **1** and **2**, respectively (distance between heavy atoms in PPa rings 1–4 and central fluorescein xanthene ring, for ring definition and plot see Figure A in S1 File). In both simulations, the chromophores quickly formed close aggregates (after ca. 16 ns for system **1** and ca. 6 ns for system **2**, Fig 4 and Figure A in S1 File).

For system **1**, a π-π-stacked contact between anthracene and PPa was first formed, and then the rest of the tail with fluorescein wound partly around PPa, and the final contacts were established after about 33.5 ns. These were stable until the end of the simulation (230.5 ns). In this complex, the fluorescein phenyl ring is π-stacked on PPa rings 1 and, to a minor extent, 3 (Figure B in S1 File), while the xanthene ring system is almost orthogonal to the PPa system. The anthracene rings are π-stacked on PPa rings 4 and possibly 3 (Figure C in S1 File). Interestingly, the amide hydrogen in the PPa spacer chain is frequently H-π-stacked on the fluorescein xanthene ring atoms (Figure D in S1 File) and, less frequently, on the anthracene ring atoms (Figure E in S1 File).

In system **2**, π-π-stacked complexes with a parallel arrangement of all three chromophores (PPa, fluorescein and anthracene linker) are preferably formed, with fluorescein and anthracene located on opposite sides of PPa. After about 6.5 ns, the chain with fluorescein winds around PPa, and a complex is formed with the central fluorescein xanthene ring clearly stacked on PPa ring 1 (Figure A in S1 File). This complex is stable almost until the end of the simulation (until 211 ns), where rings 4 and 2 dominate this contact. The fluorescein phenyl ring is also very close to PPa rings 3 and 4 for the whole simulation time (Figure B in S1 File). The central anthracene ring is nicely stacked on PPa ring 4 from 11 ns on for almost the whole simulation time, with an exception between 133 and 171 ns, where it temporarily moves away from PPa (Figure C in S1 File).

In Figure G in S1 File we analyze the root mean square fluctuations (RMSF) of the DNA residues in systems **1** and **2**, calculated over the whole simulation time, after fitting the whole trajectory on a reference structure (minimized starting structure). Some net motion (“bending”) of the double helix around two anchor regions (approximately around leading-strand residues 9 and 35) can be seen from the RMSF plots and also from visual inspection of the trajectories.

Some degree of “fraying”, i.e. opening of the terminal Watson-Crick hydrogen bonds can be observed from Figure H in S1 File for both systems **1** and **2**, but this only affects the first and last base pairs and is consistent with experimental data. [77, 78]

System **3** allows the relative orientation of anthracene linker **LA** and PPa without attached fluorescein to be investigated. This is of additional interest, as the linker conformation in this system, which does not have a negatively charged fluorescein moiety (as do systems **1** and **2**), can be studied without the strong repulsion between fluorescein and the negatively charged DNA backbone. In system **3**, the anthracene linker **LA** also forms a π-π-stacked contact with PPa after 13 ns, with the central anthracene ring close to PPa ring 4 (Figures I and J in S1 File). This is accompanied by a loss of contacts between water and the anthracene linker aromatic atoms and the PPa aromatic atoms (Figure K in S1 File).

To investigate the possible interactions between terminally attached dyes with DNA, we analyzed the simulations of **4** (with terminally attached PPa), and the system with the terminally attached anthracene linker **5**.

**Fig 5:**
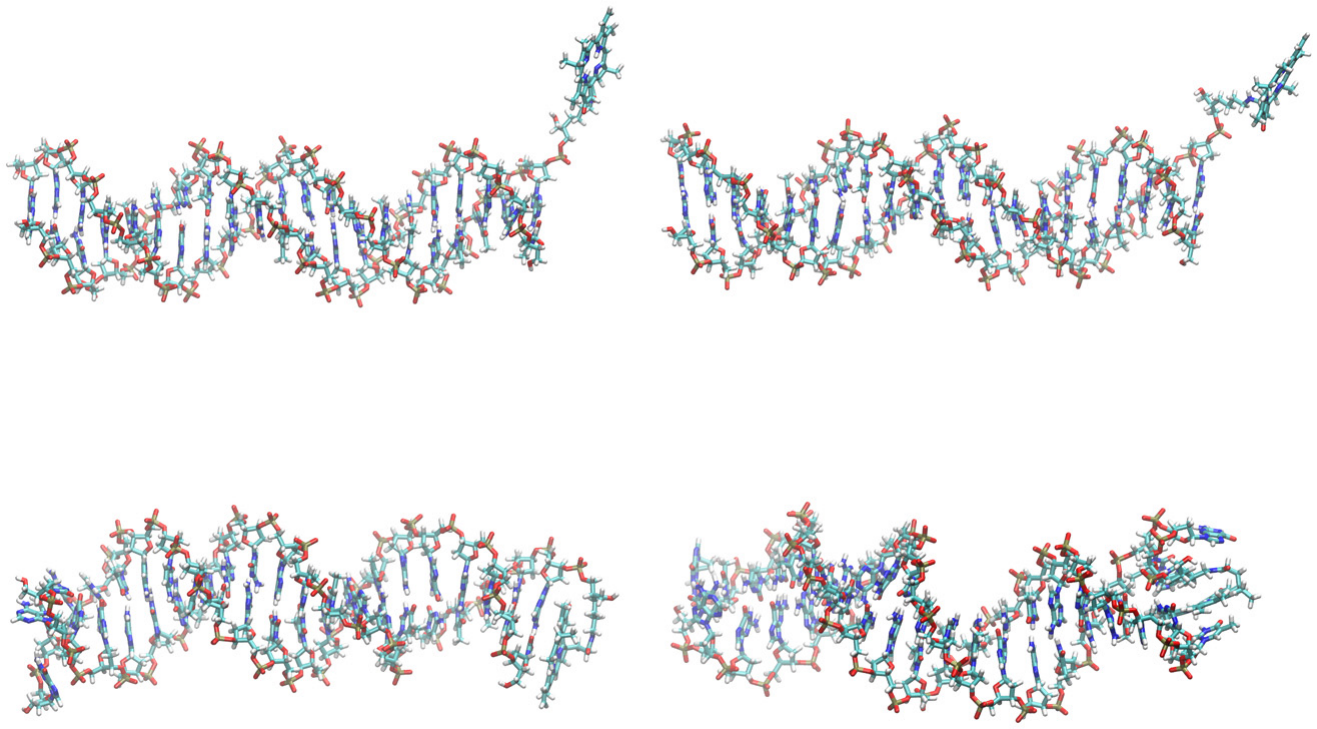
Structures of system 4 at 0.51 ns (after equilibration with harmonic restraints, above) and at 260.5 ns (below, left) and 220.5 ns (below, right), respectively. Left: Simulation at 310 K, right: simulation at 363 K. Ions and water were omitted for clarity. Figures were created using VMD. [76]

We first analyze the simulations of **4** at 310 K (37°C) and 363 K (90°C). Starting from a position dangling in the solvent, away from DNA (Fig 5), PPa forms a π-π-contact with the terminal AT base pair after 20 ns in the simulation at 310 K, with the PPa nitrogens closer to T23 than to A21 (Figures N and O in S1 File). This and the other base pairs at this terminus remain in their Watson-Crick-paired conformation for the whole simulation time, capped by PPa, while at the opposite terminus the first two base pairs are opened (“fraying”, Figure P in S1 File). In its terminus-capping conformation (310 K), the spacer chain of PPa is folded, with the carbonyl oxygen of the amide bond close to the nitrogens in the PPa ring system (Figure Q in S1 File).

In contrast to the simulation at 310 K, the three terminal base pairs at the terminus at which PPa is attached are more disrupted at 363 K, which can be seen from the analysis of the Watson-Crick hydrogen bonds (Figure P in S1 File) and also from visual inspection (Fig 5). After 7 ns, PPa is π-stacked on T23 and this contact is preserved for the whole simulation time (Figure O in S1 File). The distance from the other residue of the terminal base pair, A21 is larger, with the spacer chain of PPa between this base and the PPa ring system (Figure N in S1 File). This spacer chain is in a folded conformation, but with a slightly larger distance between the PPa nitrogens and the carbonyl oxygen of the spacer chain than in the 310 K simulation (Figure Q in S1 File). The second and the third base pair from the PPa attachment point (G20-C24 and T19-A25) are opened and no longer form Watson-Crick hydrogen bonds (Figure P in S1 File). No clear interactions between the PPa ring system and these residues are visible, with closest distances of 7–8 Å (Figure R in S1 File).

For comparison with system **4**, we simulated an oligonucleotide with identical sequence but without attached PPa at 310 K for 270.5 ns (system **4-DNA**). The system remained stable and showed some degree of bending and fraying of the terminal 1–2 base pairs (Figures S, T and U in S1 File).

**Fig 6:**
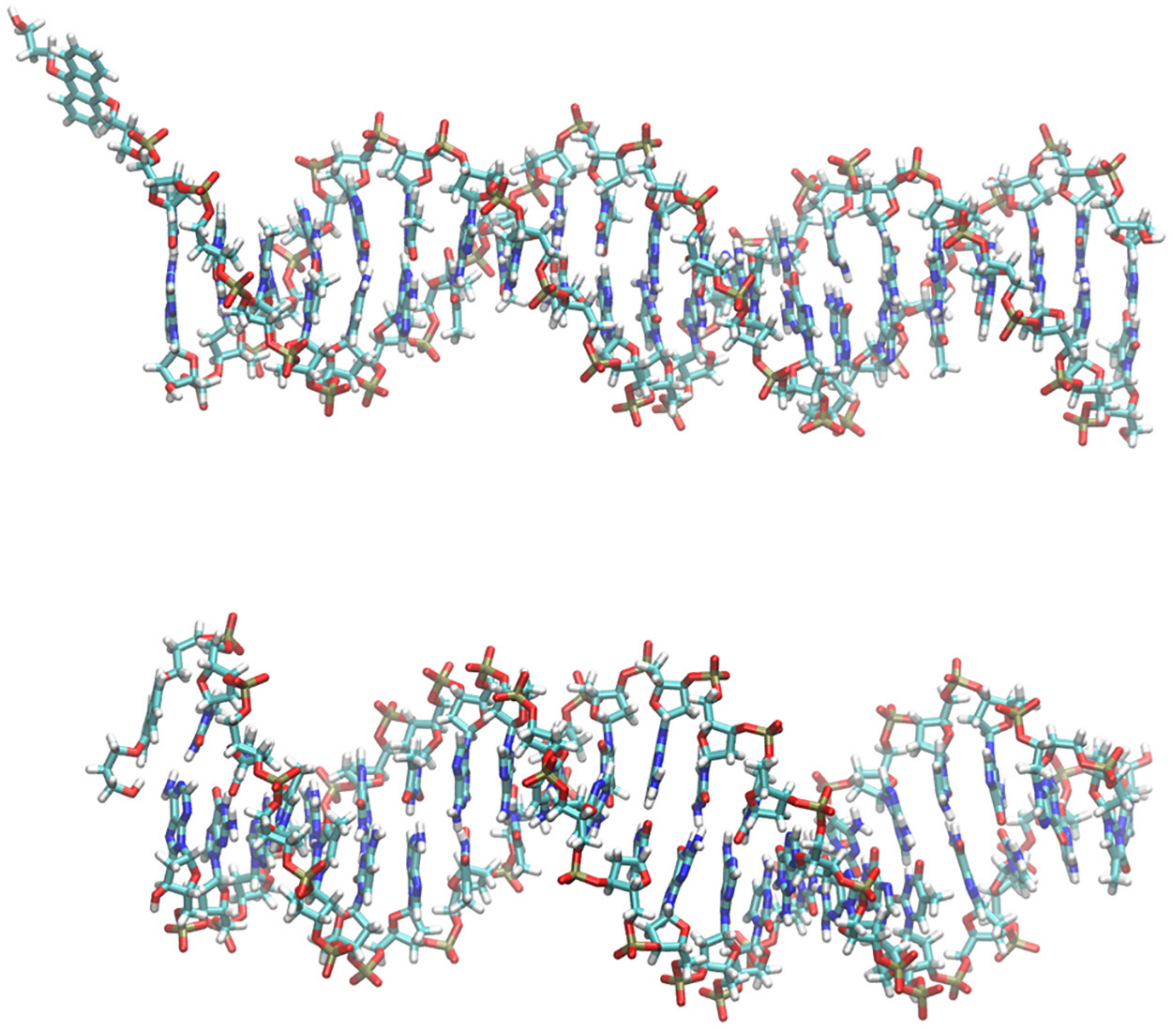
Structure of system 5 at 0.51 ns (after equilibration with harmonic restraints, above) and at 220.5 ns (below). Ions and water were omitted for clarity. Figures were created using VMD. [76]

Finally, we constructed and simulated a DNA oligonucleotide with 5’-attached anthracene linker **LA** (system **5**), which allows us to investigate how the anthracene linker interacts with the DNA fragment without negatively charged fluorescein (Fig 6). Simulation at 310 K for 220.5 ns yielded a stable structure with some fraying only at the linker-free terminus (Figure V in S1 File). Having started from a position dangling freely in solution, the anthracene linker **LA** quickly became stacked on the terminal base pair, with a close contact to the ring atoms of T2 (Figure X in S1 File).

In summary, our MD simulations show that the ring systems of the fluorophores, photosensitizers and linkers investigated preferably form π-π-stacked complexes with each other or with the base pairs at the oligonucleotide termini. This agrees well with previous findings, e.g., that indocarbocyanine dyes are stacked on terminal base pairs [37, 79] and also with the intercalation of drugs [80–84] or photosensitizers. [85] In systems **1** and **2**, which resemble “complete” nucleic acid detection systems, the potential FRET donor (fluorescein) and the potential acceptor (PPa) tend to form close contacts in the simulations (when starting from a separated geometry). It is therefore likely for the structural clusters with low donor-acceptor separation that FRET quenching is less important than contact quenching or Dexter electron transfer. We plan to investigate the exact quenching mechanisms in more detail in a future study. We also plan to extend our studies towards systems with a second dye added as internal standard or as additional fluorophore (e.g. N,N,N′,N′-tetramethylrhodamine, TAMRA). Depending on the distance between the dyes (determined by the number of base pairs between their attachment points and the length of the spacers or linkers), TAMRA fluorescence can be quenched by FRET from TAMRA to fluorescein, or by contact quenching. After irradiation and ^1^O_2_-mediated cleavage of the linker, fluorescein is released and TAMRA fluorescence can be detected. This design has the advantage that the fluorescence reporter remains bonded to the oligonucleotide and can thus be used for detection and localization. [10]

### QM/MM Calculations of System 1

#### Steady-State Absorption and Emission Spectra of System 1

**Fig 7:**
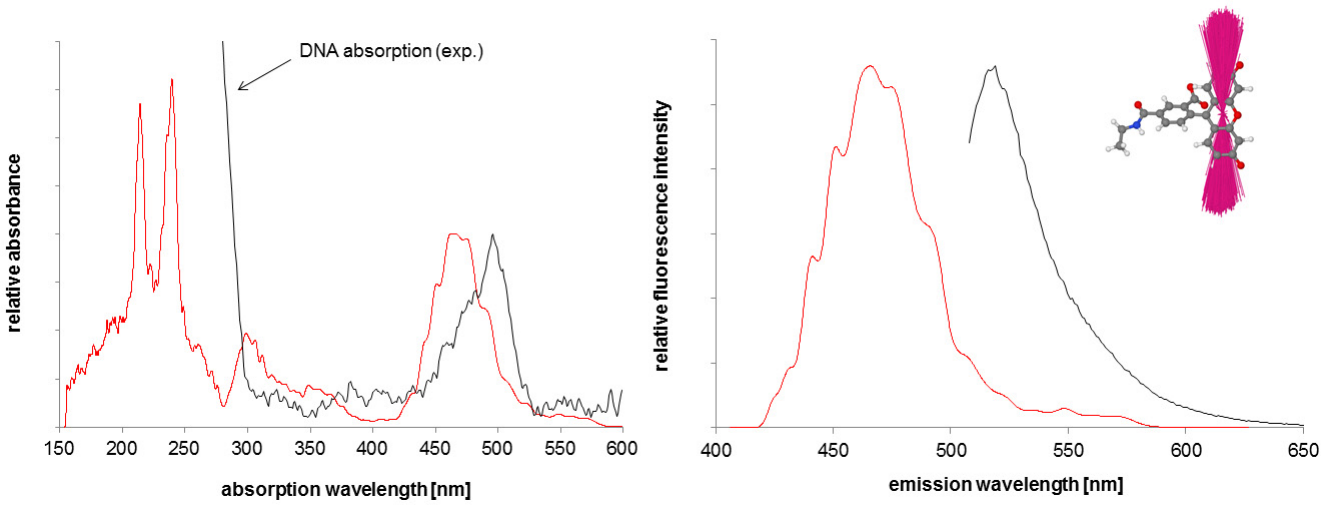
Experimental (black) and calculated (red) steady-state absorption (left) and emission spectra of fluorescein, using a subset of the MD ensemble (424 snapshots) and the QM/MM methodology described in reference [35]. **Additionally, the fitted ensemble of transition dipoles describing the S_1_ → S_0_ transition (emission spectrum) is shown (transition dipoles multiplied by a factor of 10)**. Note that the experimental spectra were recorded for systems with the chromophores attached to DNA and therefore additionally contain typical DNA absorption peaks in the UV region, while the calculated spectra resemble the situation of the chromophores in solution (due to the QM/MM formalism).

**Fig 8:**
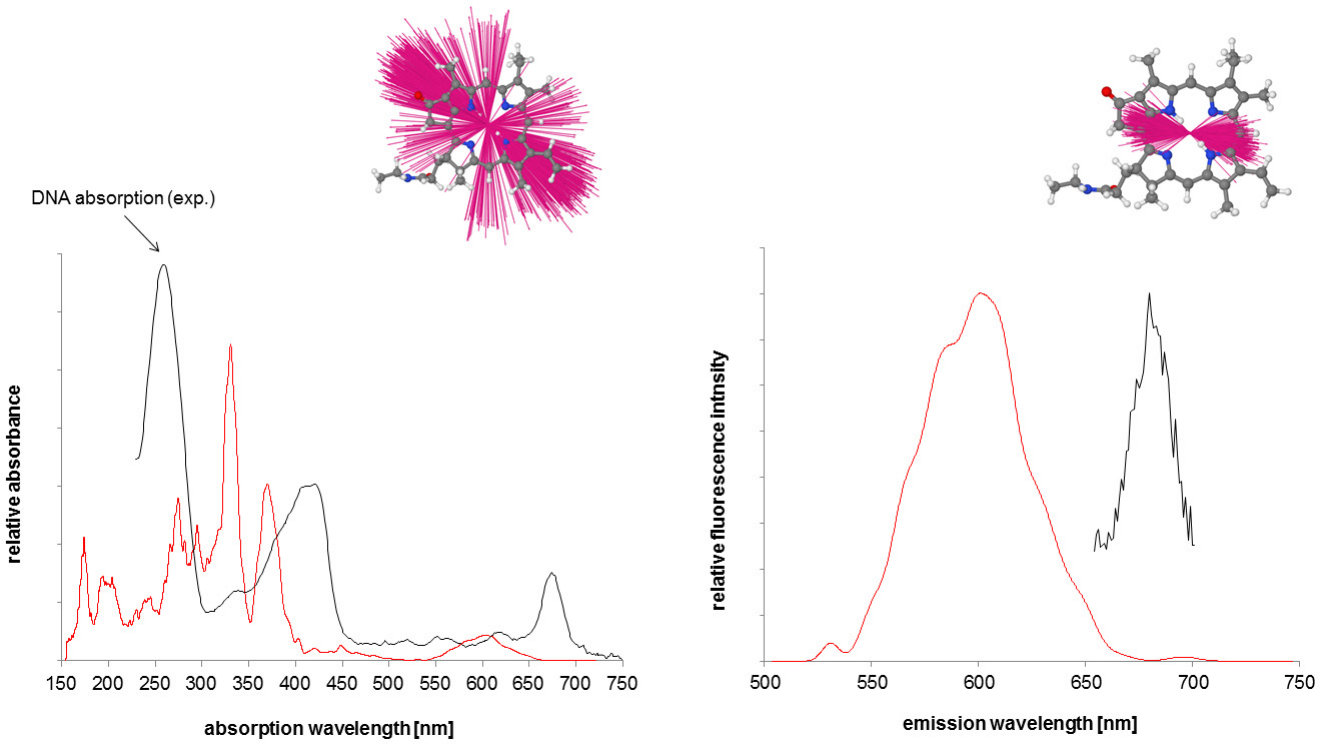
Experimental (black) and calculated (red) steady-state absorption (left) and emission spectra of PPa, using a subset of the MD ensemble (424 snapshots) and the QM/MM methodology described in reference [35]. **Additionally, fitted ensembles of transition dipoles describing the S_0_ → S_i_ transition with maximal oscillator strength (absorption spectrum) and the S_1_ → S_0_ transitions (emission spectrum) are shown (transition dipoles multiplied by a factor of 10)**. Note that the experimental spectra were recorded for systems with the chromophores attached to DNA and therefore additionally contain typical DNA absorption peaks in the UV region, while the calculated spectra resemble the situation of the chromophores in solution (due to the QM/MM formalism).

The experimental steady-state absorption and emission spectra and summed calculated vertical transitions from the simulation of system **1** are shown in Fig 7 (fluorescein) and Fig 8 (PPa). The spectra shown were calculated from a cluster with the chromophores exposed to the solvent (424 structures, cluster 2, see Figure EE in S1 File) and separated from each other, a situation which most closely resembles the dyes fluorescein and PPa in solution (due to the QM/MM formalism, see Figures Y and Z in S1 File for the cutting schemes). The experimental spectra, however, were recorded for systems with the chromophores attached to DNA-21-mers, i.e. systems like **4** and a corresponding system with fluorescein (“the right half of system **1**”) and therefore additionally contain typical DNA absorption peaks in the UV region (approx. ≤ 260 nm). The experimental fluorescence spectrum of PPa was obtained from a mixture of these two 21-mers with attached dyes. Note that relaxation effects for the excited state are not included in the calculated spectra, which therefore represent vertical transitions at the ground-state geometries (given by the simulation snapshots). [35] Fairly similar results were obtained from the semiempirical calculations with the SCRF continuum solvent model and the QM/MM calculations in which the solvent and the rest of the system were modeled as MM environment. Therefore, only the latter are shown here. Summing the calculated vertical transitions allows the band shapes of the experimental absorption spectra to be reproduced moderately well. [35] However, the simulated absorption profiles are blue-shifted by about 33 nm (fluorescein), 51 nm (PPa Soret peak) and 71 nm (largest PPa Q peak). Compared to experiment, the calculated emission peaks are blue-shifted by 53 nm (fluorescein) and 79 nm (PPa), with a good reproduction of peak shape and FWHM. Generally, we would expect calculated fluorescence profiles to consist of a narrow band at the blue end of the experimental emission peak. [35] As we only consider vertical transitions at the ground-state geometries and neglect Franck-Condon effects and excited-state relaxation completely, the summed fluorescence profiles are narrower than found in experiment. [35]. These relaxation processes would broaden and red shift the emission, so that our results are probably fairly accurate for the fluorescence within the assumptions made in our model. [35] The neglect of dispersion shifts in the theory can explain the blue shifts of the calculated absorption profile relative to the experimental spectrum. [35] A further possible explanation for the relative large blue-shift in PPa compared to fluorescein is intensity borrowing in the PPa system. Overall, the spectra are of sufficient quality for our purpose of developing a model for FRET. The difference between the fluorescein (FRET donor) emission and the PPa (FRET acceptor) absorption peak (Q peak) is similar in experiment (for the largest Q peak: ΔE = 0.55 eV) and for the calculated ensemble, where both the maximum of the ΔE distribution (not shown) and the peak difference in the spectra are approx. 0.5 eV (data for cluster 2). This is especially important as the spectral overlap *F*_*D*_*(λ)* does not explicitly exists in our model and therefore cannot be used to assess resonance between the FRET donor and the FRET acceptor. In contrast to the situation in our previous work, the calculated ΔE distribution is practically not shifted *vs*. experiment and therefore no correction is required. [35]

For the “reference” cluster 2, where the chromophores are separated from each other and are exposed to the solvent, the fitted ensembles of the transition dipoles that describe the S_0_ **→** S_i_ transition with maximal oscillator strength (absorption spectrum) and the S_1_ **→** S_0_ transitions (emission spectrum) are shown for PPa (FRET acceptor, Fig 8) and the emission transition dipoles only for fluorescein (FRET donor, Fig 7). Again, intensity borrowing in the PPa system leads to a relative large variation in the PPa absorption dipoles.

#### FRET in System 1

Experimental steady-state fluorescence spectra of system **1** reveal that FRET occurs between fluorescein and PPa: When the DNA template is added to the 21-mers carrying the anthracene linker/fluorescein and PPa, respectively, and therefore system **1** is formed, the fluorescence intensity of fluorescein (FRET donor) decreases and the intensity of PPa emission increases (FRET acceptor). These spectra are shown in Figure AA in S1 File. These results show that FRET is one important fluorescence quenching mechanism for fluorescein in the system shown, however, it is not the only possible one. Especially at lower donor-acceptor distances, alternative quenching mechanisms like contact quenching or Dexter electron transfer [86] will become more important. Investigating these will require a different QM(/MM) setup, therefore we will focus on FRET here, and investigate alternative quenching mechanisms in a subsequent study. Our approach to simulate FRET is based on the coupling of transition dipoles. It has been shown, however, that, at lower donor-acceptor distances, the dipole-dipole approximation assumed in Förster theory cannot be used. [87–89] For cases where the donor-acceptor separation is too small for dipolar coupling to be valid (system-dependent, approx. ≤ 20 Å in the case of isotropic averaging), [89] alternative approaches, e.g. ones that use the full molecular transition densities, like the transition density cube method or a linear response (LR) approach, have been developed. [38, 88, 89] Table 1 shows the results of our FRET analysis for the geometrical clusters obtained from the cluster analysis of the simulation of system **1**. As expected from visual inspection of the cluster representatives (Figures BB, CC, EE, GG-MM in S1 File), only clusters 2, 3, 4, 6, 7 and 9 have mean donor-acceptor distances larger than 20 Å (Table 1, calculated between the geometric centers of the xanthene ring atoms and the PPa ring atoms). The standard deviations calculated for the distances (Table 1) and the histograms which are additionally provided for clusters 1 and 2 (Figures DD and FF in S1 File) show that there is a certain degree of variation and that the distributions are not monomodal, indicating that the clustering process is quite challenging (although we tested several clustering approaches and only show the results for the best one). Application of Eq (1) gives the values of the orientation factor *κ*^*2*^ for each snapshot; ensemble averages are again shown in Table 1 and histograms for selected clusters in Figures DD and FF in S1 File. Again, the distributions are very anisotropic, with large standard deviations, and the ensemble averages are generally not in the range of the “isotropic value” (2/3), which would be obtained for freely rotable donors and acceptors. It is interesting, however, that the mean value of *κ*^*2*^ calculated over all snapshots in the clusters with donor-acceptor separation ≥ 20 Å (clusters 2, 3, 4, 6, 7 and 9) is actually very close to 2/3 (0.65 ± 0.70), but this may be fortuitous. Nevertheless, it indicates that the linkers are long and flexible enough to provide reasonable sampling of relative donor-acceptor geometries. Application of Eq (2), i.e. modulation of *κ*^*2*^ with *r*^*-6*^, gives the distributions of relative FRET rate constants *k*_*T*,*rel*_, which are again broad (Figures DD and FF in S1 File) with large standard deviations (Table 1), and span several orders of magnitude, mainly caused by the large differences in the distances between the clusters. We further calculated Förster radii for the individual snapshots (Eq 3) and report the ensemble averages. These are about 28 Å (30 Å if only clusters with a donor-acceptor separation > 20 Å are considered), with large standard deviations. We also show ensemble averages of the FRET efficiencies calculated for each snapshot (Eq 5), these are expected to be 50% at a donor-acceptor separation equal to the Förster radius, and are much higher at closer distances and much lower at larger separations. The ensemble average of the FRET efficiencies is 0.50 ± 0.33 if only the clusters with donor-acceptor distance > 20 Å are considered (where the dipolar approximation is valid). This value agrees with the experimental FRET efficiency determined for this system (ca. 0.3, Figure AA in S1 File). Overall, our simulations show that the different relative donor-acceptor geometries possess distinctive FRET efficiencies and rates, both caused by distance and orientation effects. It is also interesting to look at the averages of the Einstein coefficients describing donor fluorescence *(kr)*; these also show variation between the clusters and possibly reflect the fact that in clusters with direct donor-acceptor contact the larger charges in the MM part close to the QM/MM interface lead to smaller fluorescence compared to the case in which the chromophore is surrounded by water only. In a subsequent study, we plan to develop a more complete model of fluorescence kinetics, as in our previous work on protein fluorescence. [35]

**Table 1:** Geometrical clusters obtained from the MD simulation of system 1, mean (ensemble average) distances between chromophores involved in FRET (xanthene ring system in fluorescein and ring system of PPa), mean orientation factor, mean of the Förster radii (calculated for the individual snapshots), mean FRET efficiency, mean relative FRET rate constant, mean radiative rate constant (Einstein coefficient) of the donor. Errors are given as standard deviations. Clusters with donor-acceptor distance > 20 Å are highlighted in boldface.

Cluster	#Snaphots	Fraction	< r > [Å]	±	<κ^2^>	±	< R_0_ > [Å]	±	< E_FRET_ >	±	< k_T,rel_ > [10^−9^ s^-1^]	±	< k_r_ > [10^7^ s^-1^]	±
0	20530	0.89	9.71	1.48	0.42	0.53	27.53	7.98	0.97	0.12	848.46	1870.25	8.25	8.50
1	705	0.03	13.36	4.43	0.91	0.95	31.76	8.88	0.93	0.19	1003.19	2423.83	8.59	8.65
**2**	**424**	**0.02**	**29.73**	**4.68**	**1.05**	**0.88**	**33.92**	**7.76**	**0.65**	**0.28**	**2.36**	**3.52**	**18.37**	**6.35**
**3**	**383**	**0.02**	**32.70**	**8.03**	**0.48**	**0.53**	**28.62**	**7.86**	**0.41**	**0.34**	**2.30**	**8.62**	**16.84**	**8.05**
**4**	**304**	**0.01**	**28.04**	**3.14**	**0.39**	**0.43**	**27.66**	**7.60**	**0.49**	**0.31**	**1.09**	**1.60**	**9.04**	**8.69**
5	206	0.01	10.57	1.23	0.45	0.53	28.09	7.98	0.96	0.16	385.78	522.02	10.64	8.76
**6**	**185**	**0.01**	**37.10**	**4.20**	**0.54**	**0.45**	**30.32**	**6.78**	**0.28**	**0.19**	**0.24**	**0.35**	**13.31**	**8.47**
**7**	**132**	**0.01**	**23.16**	**3.12**	**0.76**	**0.82**	**29.87**	**9.76**	**0.68**	**0.34**	**7.78**	**12.10**	**4.98**	**6.48**
8	80	0.00	17.78	2.29	0.72	0.91	29.34	9.53	0.84	0.27	29.69	50.28	6.34	7.70
**9**	**51**	**0.00**	**26.96**	**3.34**	**0.27**	**0.31**	**25.97**	**6.86**	**0.47**	**0.30**	**0.82**	**0.94**	**10.24**	**8.64**
0–9	23000	1.00	11.21	5.63	0.46	0.57	27.85	8.09	0.93	0.19	791.06	1830.00	8.65	8.65
**2,3,4,6,7,9**	**1479**	**0.06**	**30.44**	**6.47**	**0.65**	**0.70**	**30.20**	**8.22**	**0.50**	**0.33**	**2.23**	**6.29**	**14.02**	**8.90**

## Conclusions and Outlook

We have developed and tested a protocol to simulate DNA-dye conjugates and have compared the simulations to experimental work conducted on the same systems. Our results show that the parameterization and simulation protocol is robust enough to extend future studies to a much wider range of oligonucleotides, dyes and linkers. Future work will investigate alternative designs, e.g. with a second dye added as internal standard or additional fluorophore. [10] Additionally, we will use our results to optimize the linkers, spacers and chromophores in the experimental detection systems, and their ideal relative orientations, i.e. the optimal number of base pairs between the dyes and the optimal spacer length, further. While initial experiments and calculations have been performed with DNA-dye systems (due to the more reliable force fields available for DNA and the lower costs in the experimental part of the study), future work will switch towards RNA systems, e.g. to detect, localize and visualize specific mRNA sequences *in vitro* and *in vivo*. The ultimate goal is to enhance sensitivity to be able to detect a single RNA sequence in a cell, and to increase sequence specificity to allow single nucleotide discrimination. For applications in live cells, it is further important to enhance stability towards nucleases and to increase membrane permeability. The former can be achieved by using 2’-OMe-substituted RNAs or phosphorothioate DNAs or RNAs, in which a non-bridging oxygen in the phosphate backbone is replaced by sulphur. Membrane permeability can, e.g., be enhanced by conjugation to carbon allotropes, like single-walled carbon nanotubes (SWNT) or graphene oxide (GO). [90–96]

The structural ensembles obtained from the MD-simulations of these new systems will be used to calculate fluorescence emission and absorption spectra and to investigate possible quenching pathways (e.g., FRET and contact quenching) in more detail, using our previously published QM/MM approach. [35] We also currently perform computational melting experiments [97] for oligonucleotide fragments with terminally attached dyes (**4** and **5** and their pure DNA analogues), which will provide additional insight in the interaction between the terminally attached dyes with DNA and its consequences for duplex stability and melting. We further plan to use metadynamics [98] to investigate the relative interaction strengths of these terminally stacked chromophores.

## Supporting Information

S1 File (PDF file containing additional figures and some remarks on nucleic acid force fields). Figures A-H: Measurements and analyses for systems **1** and **2**; Figures I-L: Visualization, measurements and analyses for system **3**; Figures M-R: Measurements, analyses and additional visualizations for system **4**; Figures S-U: Visualizations, measurements and analyses for system **4-DNA**; Figures V-X: Measurements and analyses for system **5**; Figures Y-Z: Cutting scheme for the QM/MM calculations; Figure AA: Experimental evidence for FRET in system **1**; Figures BB-MM: Representative snapshots of the clusters obtained for system **1**, and FRET quantities obtained from the QM/MM calculations (for clusters 1 and 2); Nucleic acid force fields.
(PDF)
