# Supplementary material for: DNA-Dye-Conjugates: Conformations and Spectra of Fluorescence Probes
Source: PLoS One. 2016 Jul 28;11(7):e0160229. doi: 10.1371/journal.pone.0160229 (PMC4965132; doi:10.1371/journal.pone.0160229)
Supplement: S1 File — Figures A-H: Measurements and analyses for systems 1 and 2; Figures I-L: Visualization, measurements and analyses for system 3; Figures M-R: Measurements, analyses and additional visualizations for system 4; Figures S-U: Visualizations, measurements and analyses for system 4-DNA; Figures V-X: Measurements and analyses for system 5; Figures Y-Z: Cutting scheme for the QM/MM calculations; Figure AA: Experimental evidence for FRET in system 1; Figures BB-MM: Representative snapshots of the clusters obtained for system 1, and FRET quantities obtained from the QM/MM calculations (for clusters 1 and 2); Nucleic acid force fields. (PDF) [file pone.0160229.s001.pdf]

Supporting Information

DNA-Dye-Conjugates: Conformations and Spectra of Fluorescence Probes

Frank R. Beierlein,[1,2] Miguel Paradas Palomo,[1,3,#a] Dmitry I. Sharapa,[1,2] Oleksii Zozulia,[3] Andriy Mokhir[3] and Timothy Clark[1,2]*

[1] Friedrich-Alexander-Universität Erlangen-Nürnberg, Department of Chemistry and Pharmacy, Computer-Chemistry-Center and Interdisciplinary Center for Molecular Materials, Nägelsbachstr. 25, 91052 Erlangen, Germany

[2] Friedrich-Alexander-Universität Erlangen-Nürnberg, Cluster of Excellence Engineering of Advanced Materials, Nägelsbachstr. 49b, 91052 Erlangen, Germany

[3] Friedrich-Alexander-Universität Erlangen-Nürnberg, Department of Chemistry and Pharmacy, Organic Chemistry II, Henkestr. 42, 91054 Erlangen, Germany

[#a] Current Address: Henkel-UAB Programme, Edifici Eureka, Campus UAB, 08193 Bellaterra, Barcelona, Spain

Supporting Information

Figures A-H: Measurements and analyses for systems **1** and **2** 3

Figures I-L: Visualization, measurements and analyses for system **3** 11

Figures M-R: Measurements, analyses and additional visualizations for system **4** 15

Figures S-U: Visualizations, measurements and analyses for system **4-DNA** 21

Figures V-X: Measurements and analyses for system **5** 24

Figures Y-Z: Cutting scheme for the QM/MM calculations 27

Figure AA: Experimental evidence for FRET in system **1** 29

Figures BB-MM: Representative snapshots of the clusters obtained for system **1**, and FRET quantities obtained from the QM/MM calculations (for clusters 1 and 2) 30

Nucleic acid force fields 42

References 43

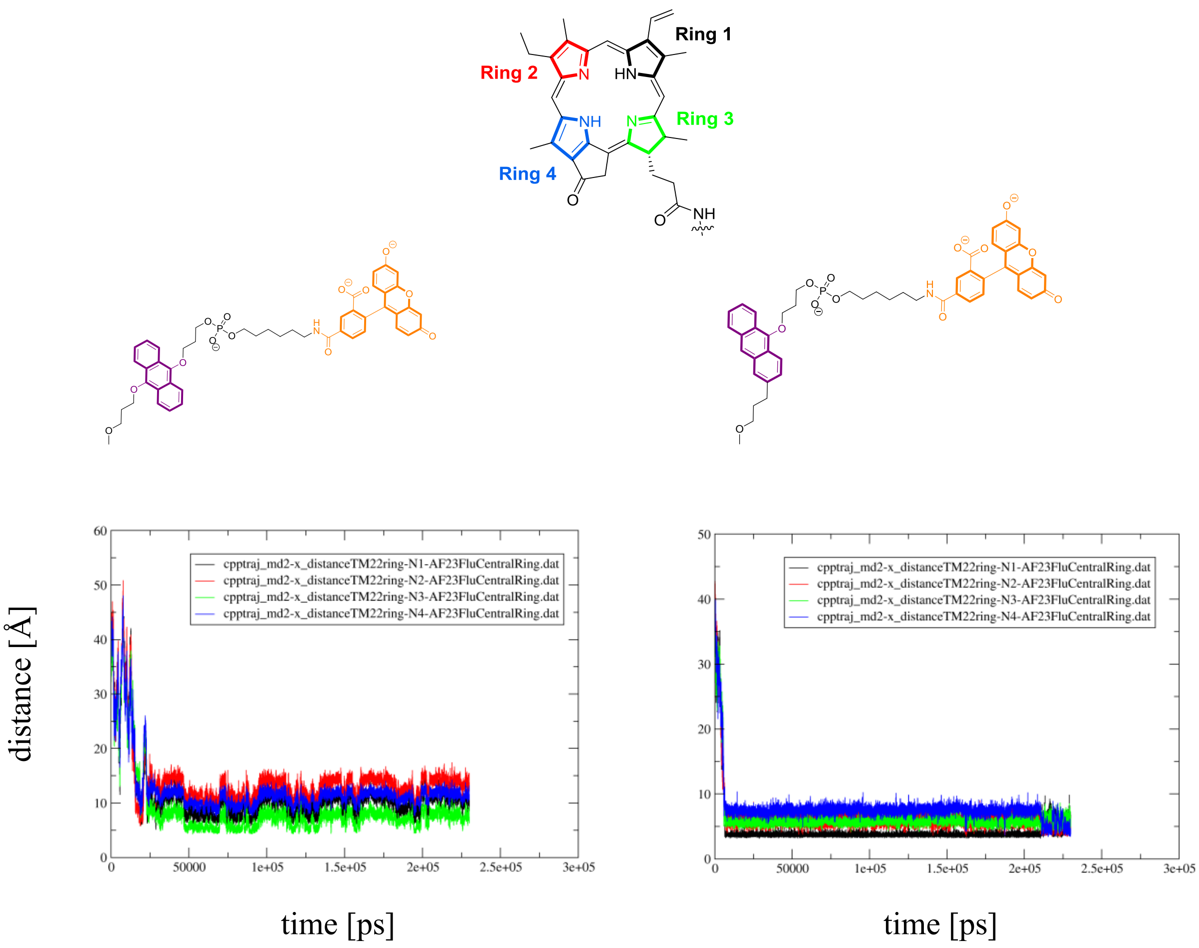

Fig. A. Above: definition of ring systems used for the measurements. Below: distance between atoms in PPa rings 1-4 and central fluorescein xanthene ring. Below, left: system **1**, below, right: system **2**.

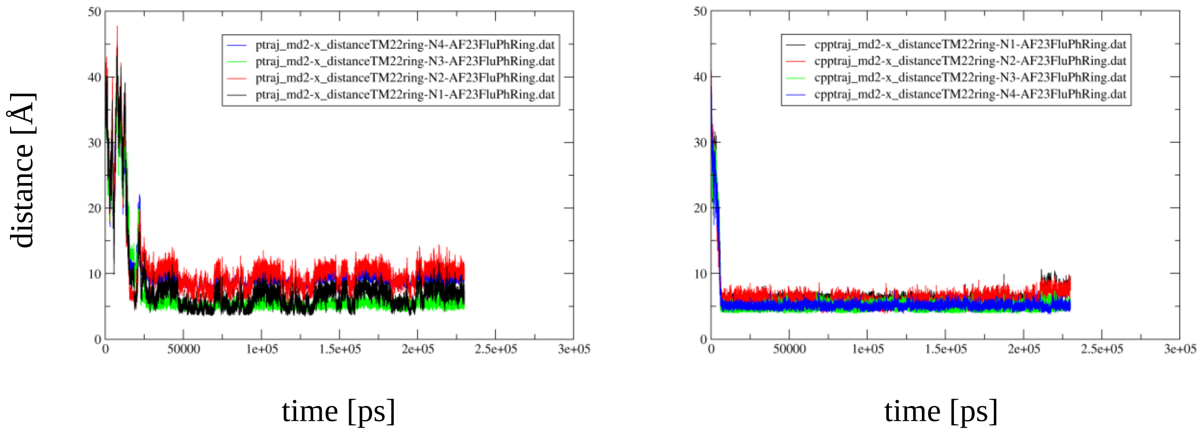

Fig. B. Distance between atoms in PPa rings 1-4 and fluorescein phenyl ring. Left: system **1** right: system **2**. For ring definitions, see Fig. .

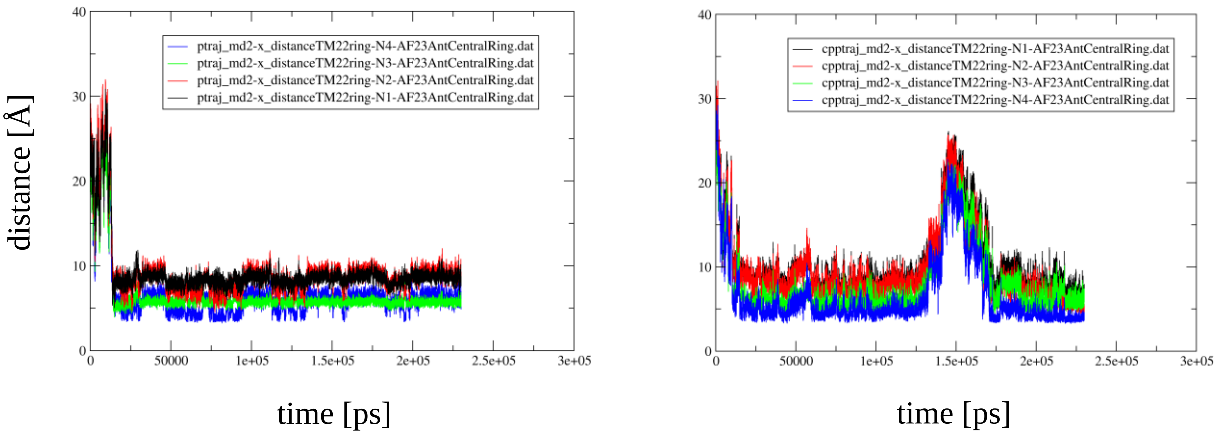

Fig. C. Distance between atoms in PPa rings 1-4 and central anthracene ring. Left: system **1**, right: system **2**. For ring definitions, see Fig. .

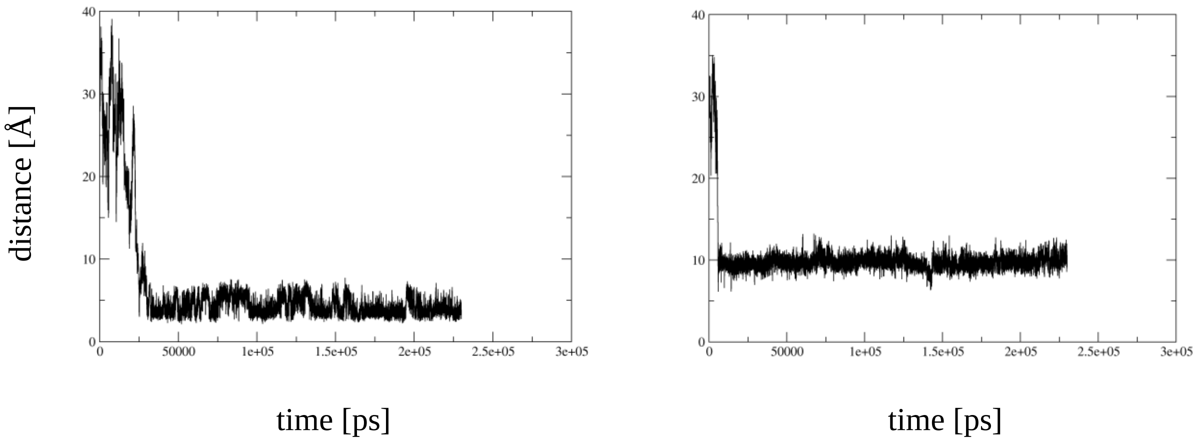

Fig. D. Distance between amide hydrogen (H45) in PPa and fluorescein xanthene ring atoms. Left: system **1**, right: system **2**.

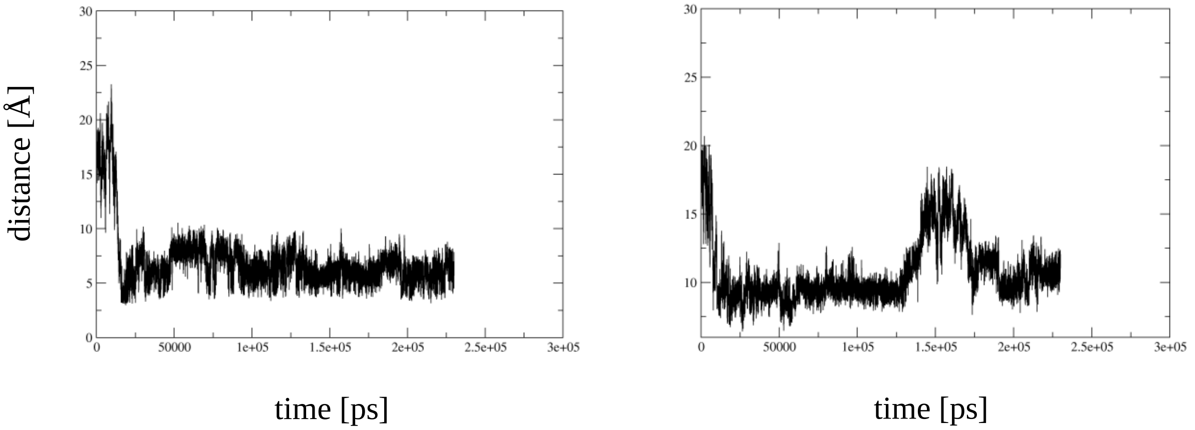

Fig. E. Distance between amide hydrogen (H45) in PPa and anthracene ring atoms. Left: system **1**, right: system **2**.

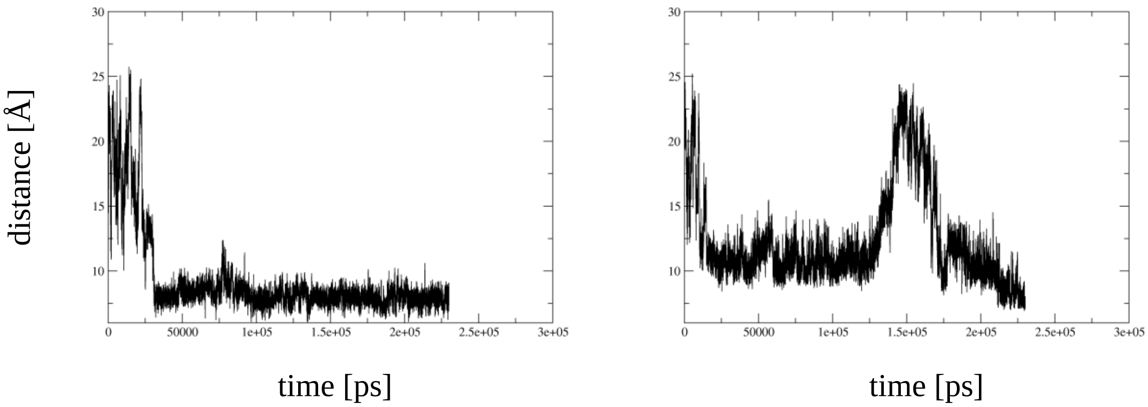

Fig. F. Distance between atoms in anthracene rings and fluorescein xanthene rings. Left: system **1**, right: system **2**.

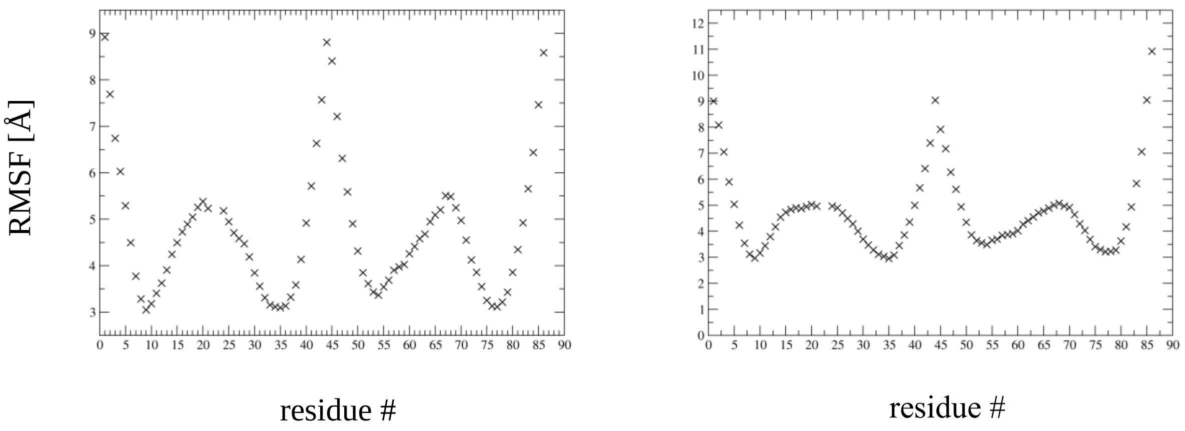

Fig. G. Root mean square fluctuations (RMSF), calculated for the DNA heavy atoms (after fitting the whole system on the minimized starting structure, using the DNA backbone atoms for fitting) vs. residue number. Left: system **1**, right: system **2**. The oligonucleotide fragments in 5' to 3' order are: base pair (BP) 1-21 with covalently attached PPa (residue 22, 3'-terminal), anthracene-linker-fluorescein (residue 23, 5'-terminal) covalently bonded to BP 24-44, and BP 45-86 (target strand).

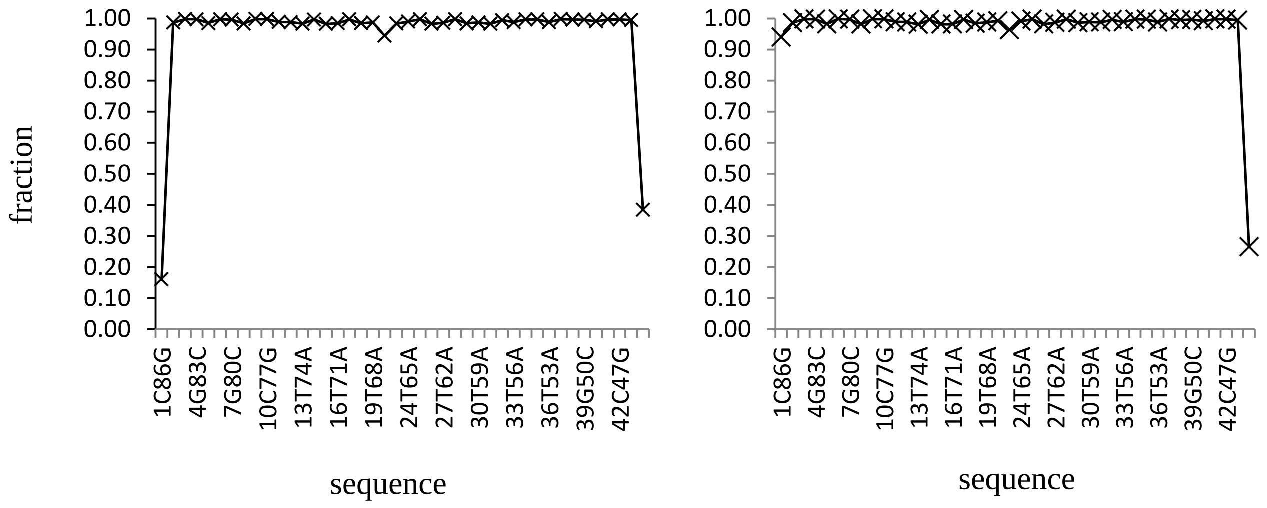

Fig. H. Fraction of canonical Watson-Crick-H-bonds formed (compared to the maximal possible number) vs. sequence, averaged over the whole simulation time. Left: system **1**, right: system **2**. 1.0 indicates that all possible Watson-Crick hydrogen bonds are formed (2 for AT and 3 for GC), 0.0 that none are formed. Sequence information is given using the format "residue number in forward strand, base, residue number in reverse strand, base".

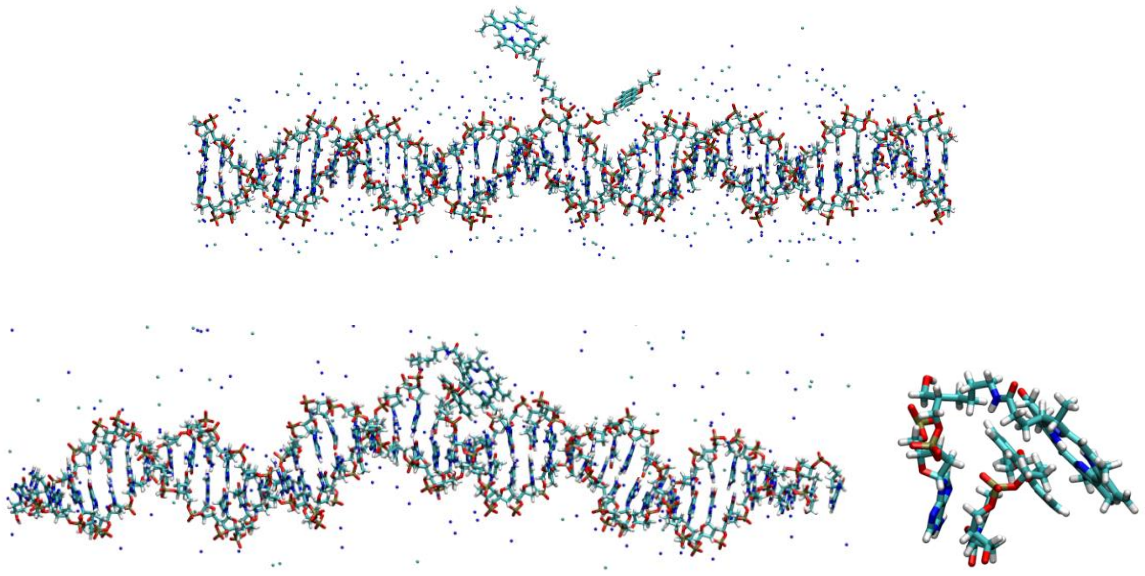

Fig. I. Structures of system **3** at 0.51 ns (after equilibration with harmonic restraints, above) and at 100.5 ns (below, left). Below, right: additional view of the chromophores at 100.5 ns. CPK sticks: DNA, linkers/spacers and chromophores. Small spheres: Na^+ (blue) and Cl^- (cyan) ions. Figures were created using VMD.[1]

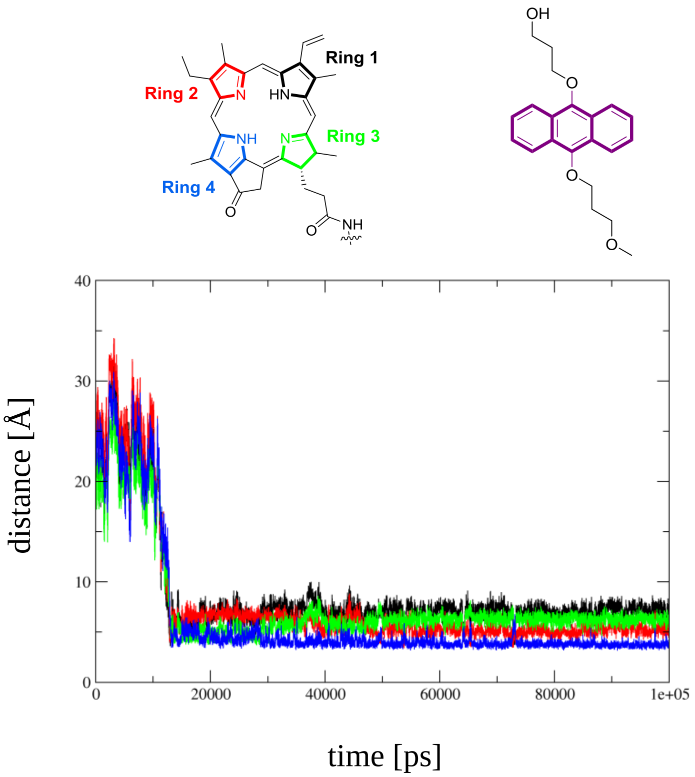

Fig. J. Distance between atoms in PPa rings 1-4 and central anthracene ring in system **3**.

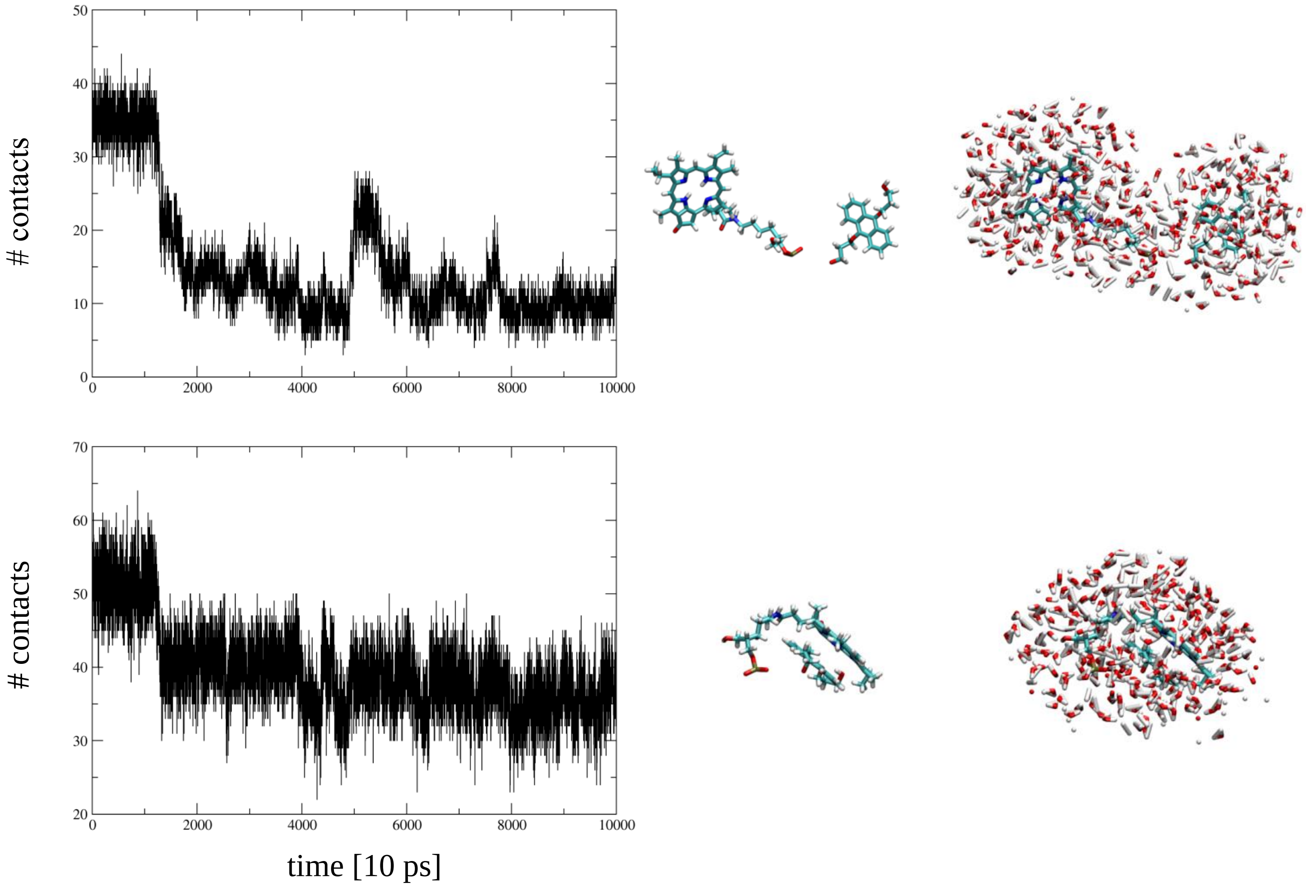

Fig. K. System **3**: Number of water oxygen contacts between 5 and 7 Å around anthracene linker aromatic heavy atoms (above, left) and around PPa aromatic heavy atoms (below, left) and views of the separated (above, right) and stacked (below, right) chromophores.

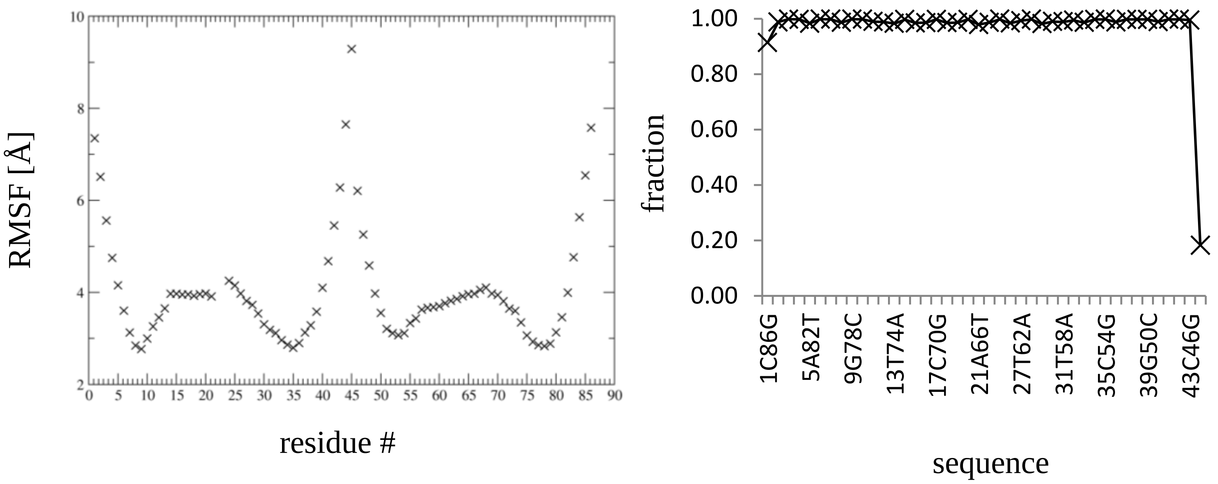

Fig. L. System **3**, simulation at 310 K: Left: RMSF, calculated for DNA heavy atoms, vs. residue number. Right: fraction of canonical Watson-Crick-H-bonds formed (compared to the maximal possible number) vs. sequence (here, sequence information is given using the format "residue number in forward strand, base, residue number in reverse strand, base").

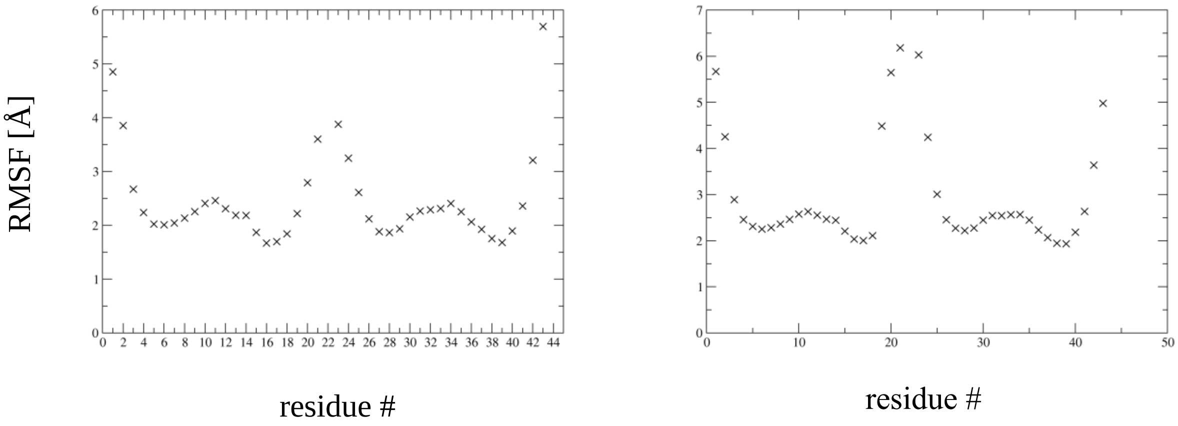

Fig. M. System **4**: RMSF, calculated for DNA heavy atoms, vs. residue number. Left: simulation at 310 K, right: simulation at 363 K.

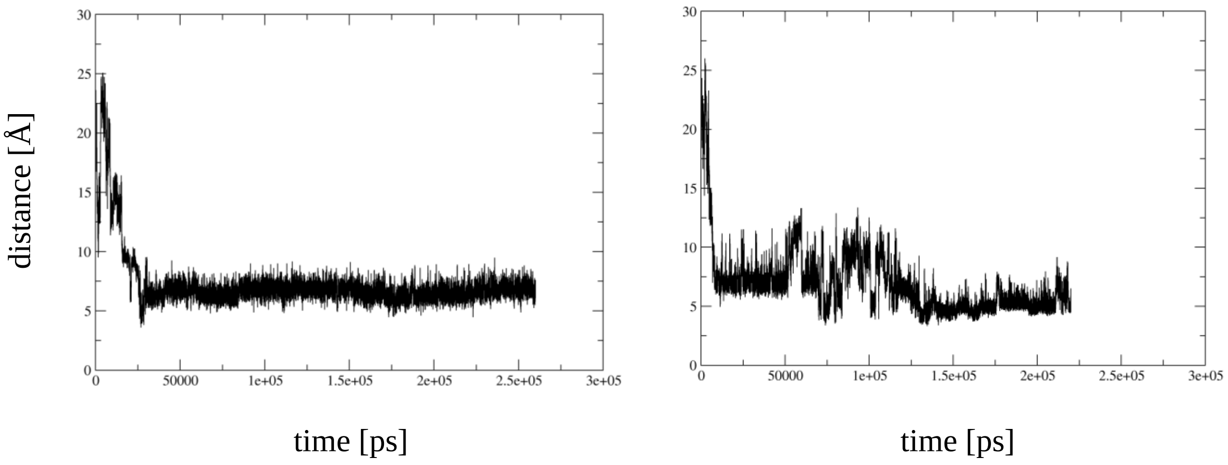

Fig. N. System **4**: Distance between PPa N1,N2,N3,N4 atoms and A21 ring atoms. Left: 310 K (mean calculated from 26.4-260.5 ns: 6.59 ± 0.64 Å), right: 363 K.

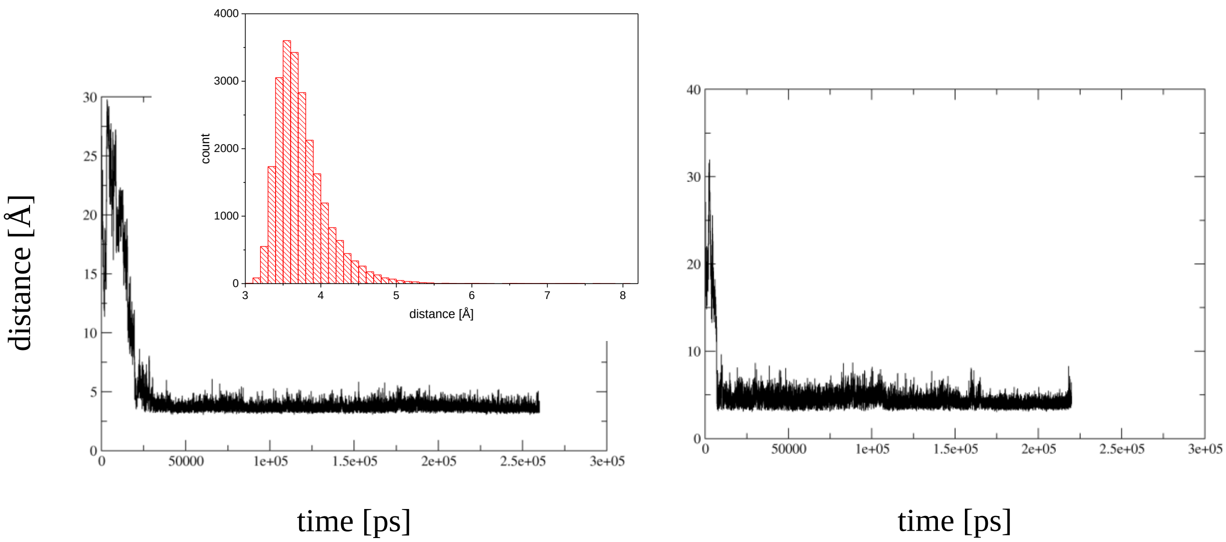

Fig. O. System **4**: Distance between PPa N1,N2,N3,N4 atoms and T23 ring atoms. Left: 310 K (mean calculated from 26.4-260.5 ns: 3.75 ± 0.37 Å, histogram bin width 0.1 Å), right: 363 K. The average base pair step rise in this sequence is 3.37 Å (helical rise: 3.13 Å).

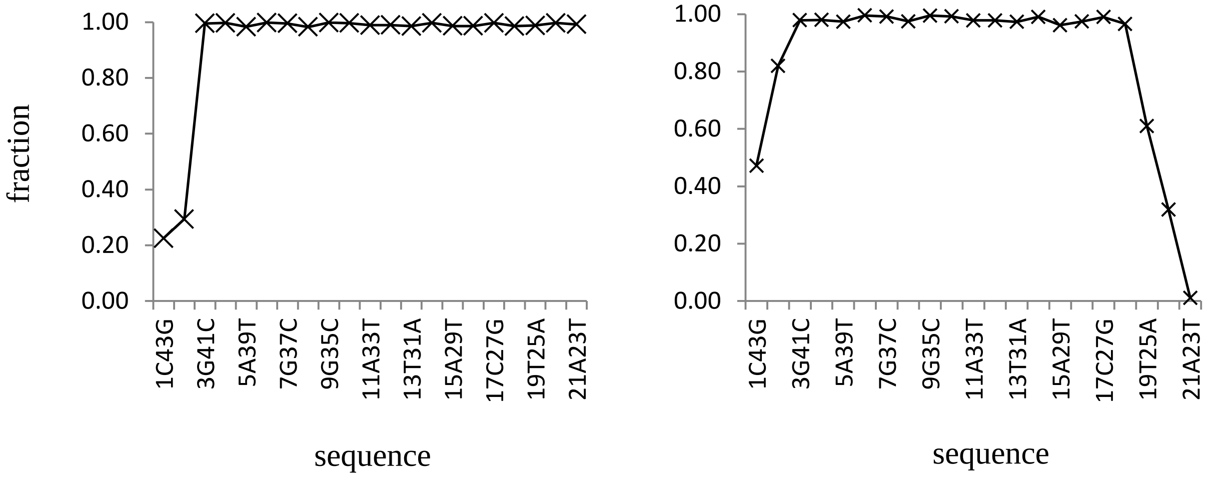

Fig. P. System **4**: Fraction of canonical Watson-Crick-H-bonds formed (compared to the maximal possible number) vs. sequence. Left: 310 K, right: 363 K. Sequence information is given using the format “residue number in forward strand, base, residue number in reverse strand, base”.

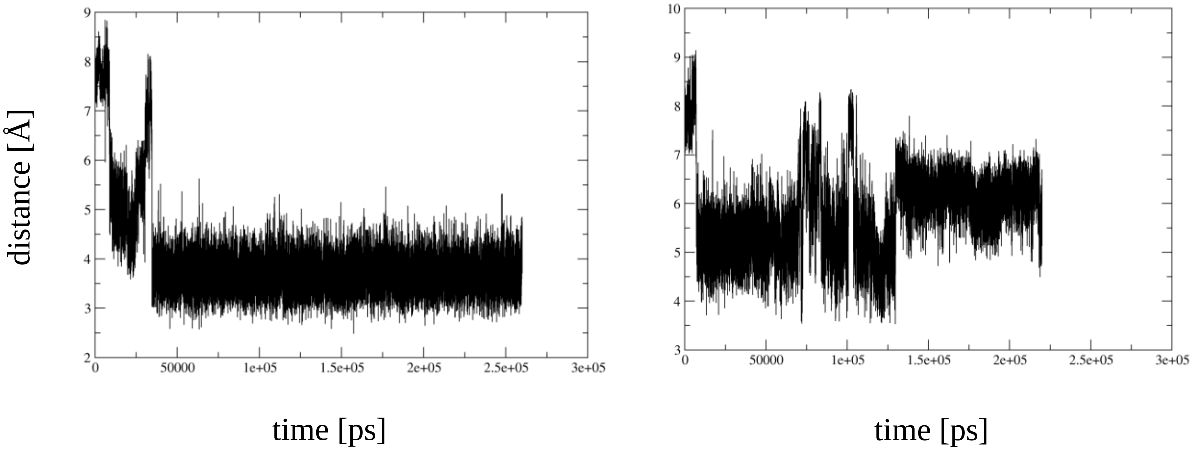

Fig. Q. System **4**: Distance between PPa N1,N2,N3,N4 atoms and the carbonyl oxygen of the amide bond of the PPa spacer. Left: 310 K, right: 363 K.

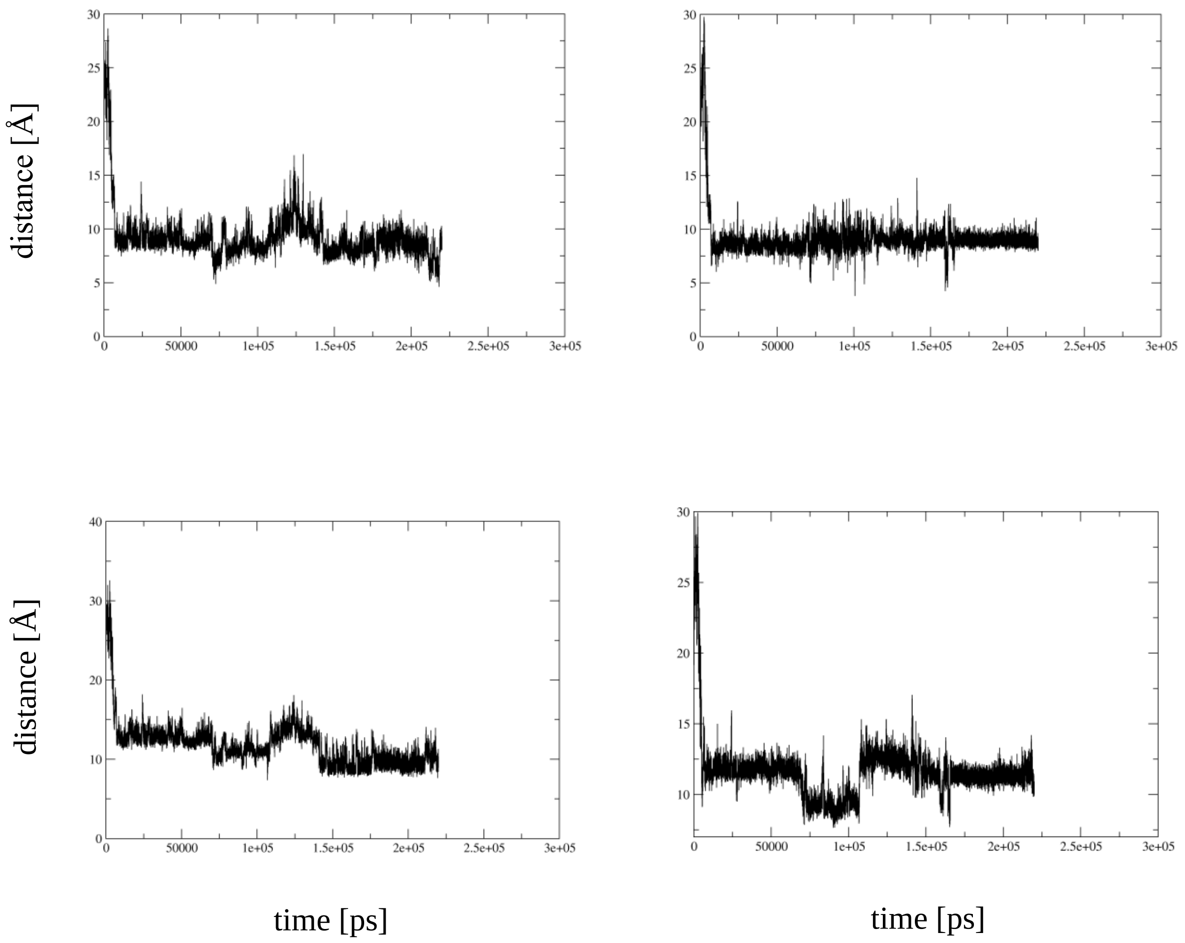

Fig. R. System **4**, simulation at 363 K: Distance between PPa N1,N2,N3,N4 atoms and G20 (above, left), C24 (above, right), T19 (below, left) and A25 ring atoms (below, right).

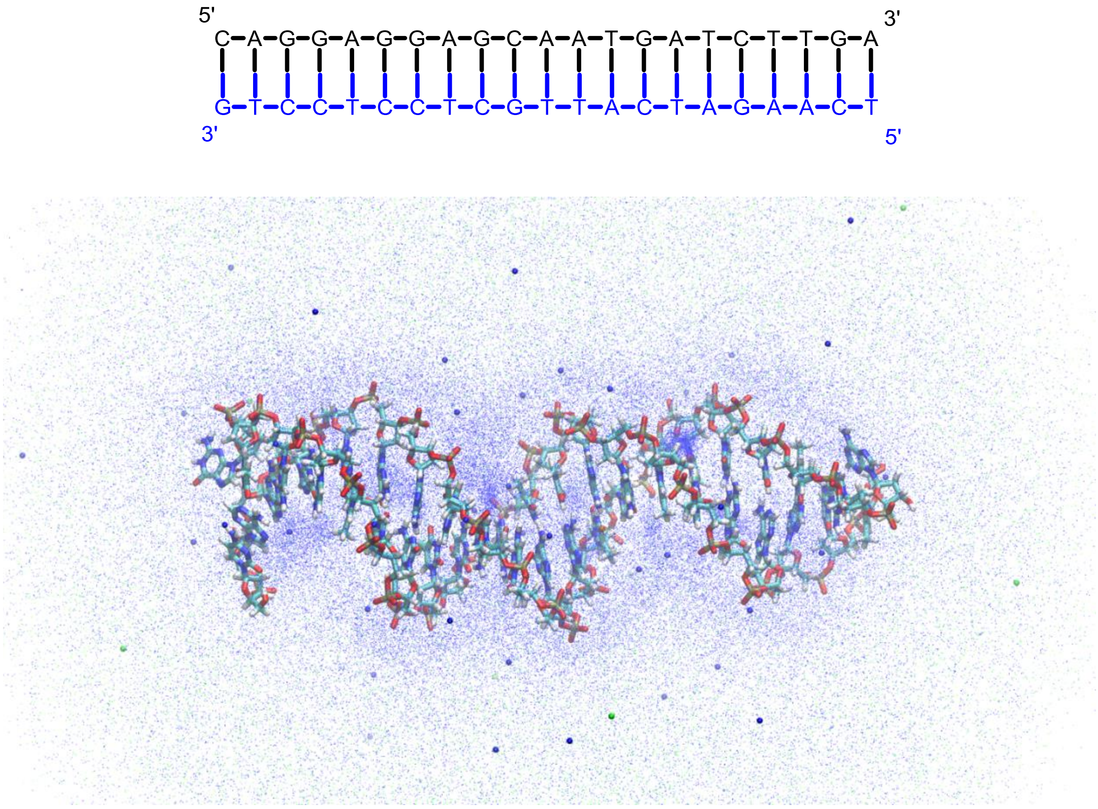

Fig. S. Structure of system **4-DNA** (pure DNA) at 270.5 ns, simulation at 310 K. CPK sticks: DNA, linkers/spacers and chromophores. Small spheres: Na^+ (blue) and Cl^- (green) ions. Additionally, for the ions, every 10th snapshot is shown for the interval 0.5-270.5 ns (blue: Na^+, green: Cl^-). Figures were created using VMD.[1]

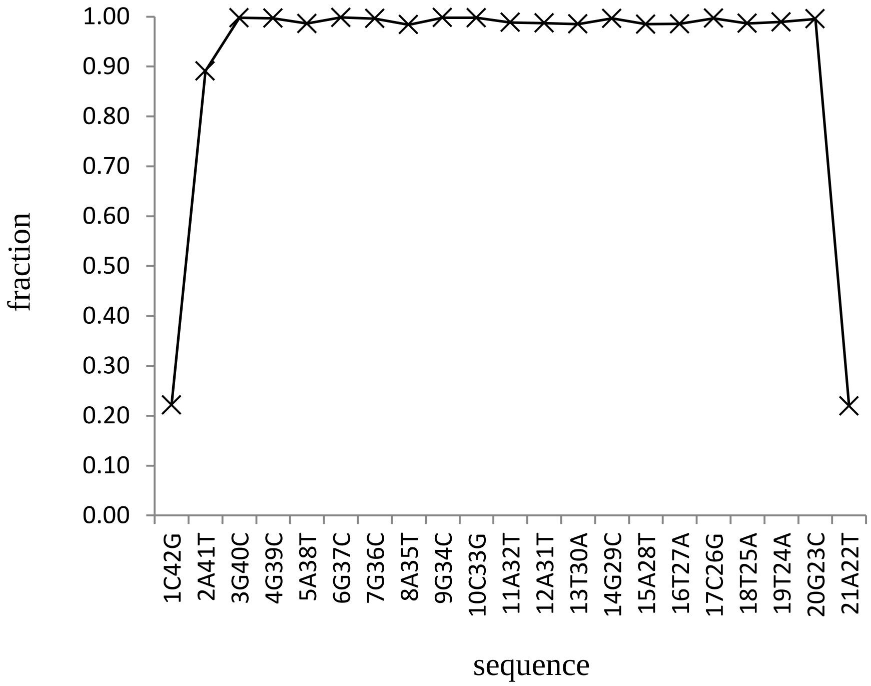

Fig. T. System **4-DNA**, simulation at 310 K: fraction of canonical Watson-Crick-H-bonds formed (compared to the maximal possible number) vs. sequence. Sequence information is given using the format "residue number in forward strand, base, residue number in reverse strand, base".

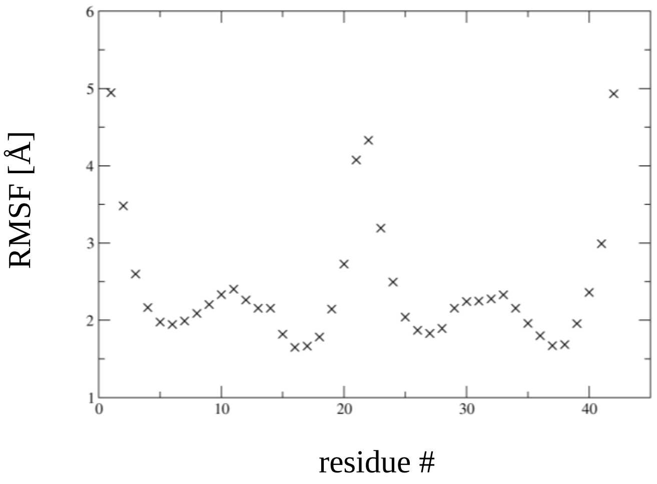

Fig. U. System **4-DNA**, simulation at 310 K: RMSF, calculated for DNA heavy atoms, vs. residue number.

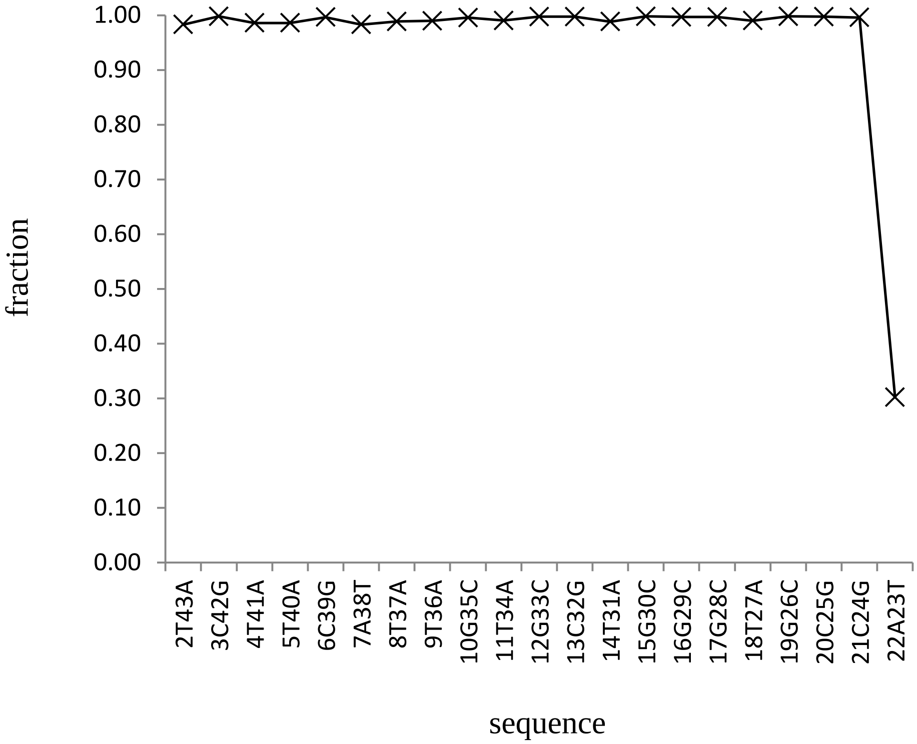

Fig. V. System **5**, simulation at 310 K: fraction of canonical Watson-Crick-H-bonds formed (compared to the maximal possible number) vs. sequence. Sequence information is given using the format "residue number in forward strand, base, residue number in reverse strand, base".

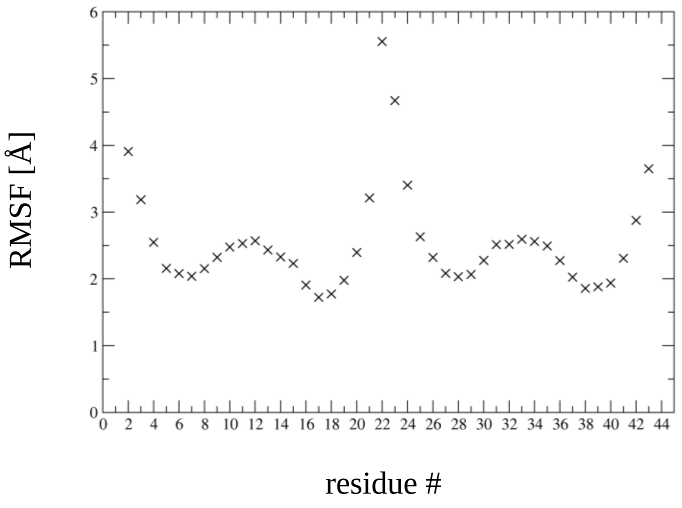

Fig. W. System **5**, simulation at 310 K: RMSF calculated for DNA heavy atoms, vs. residue number.

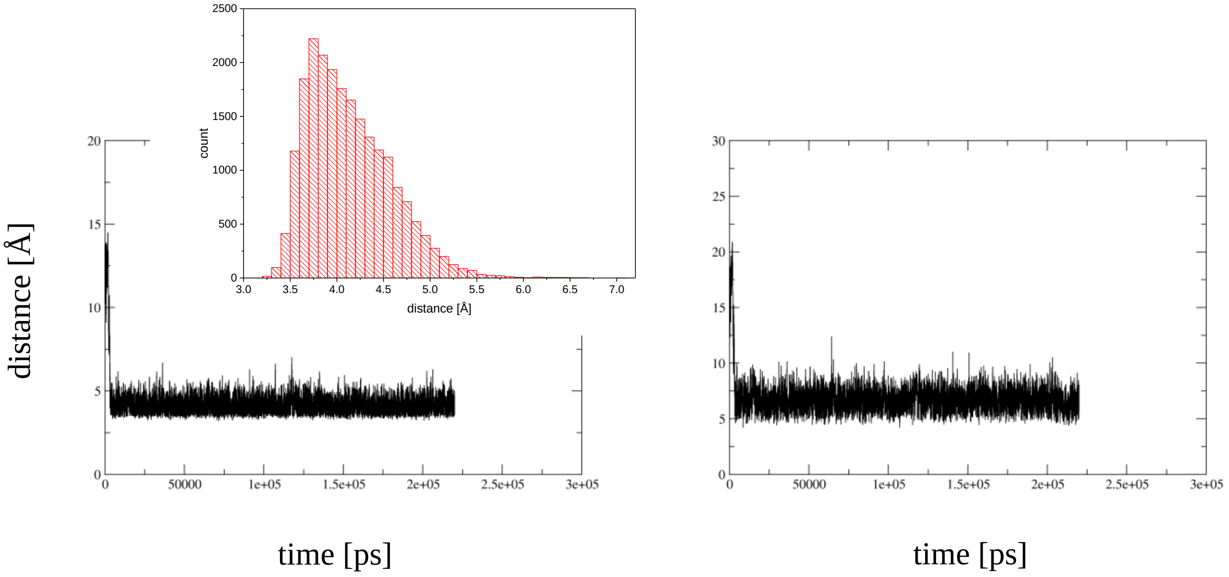

Fig. X. System **5**, simulation at 310 K: Distance between anthracene linker **LA** ring atoms and T2 (left, mean calculated from 4.0-220.5 ns: 4.13 ± 0.45 Å, histogram bin width 0.1 Å), and A43 ring atoms (right, mean calculated from 4.0-220.5 ns: 6.69 ± 0.91 Å). The average base pair step rise in this sequence is 3.31 Å (helical rise: 3.07 Å).

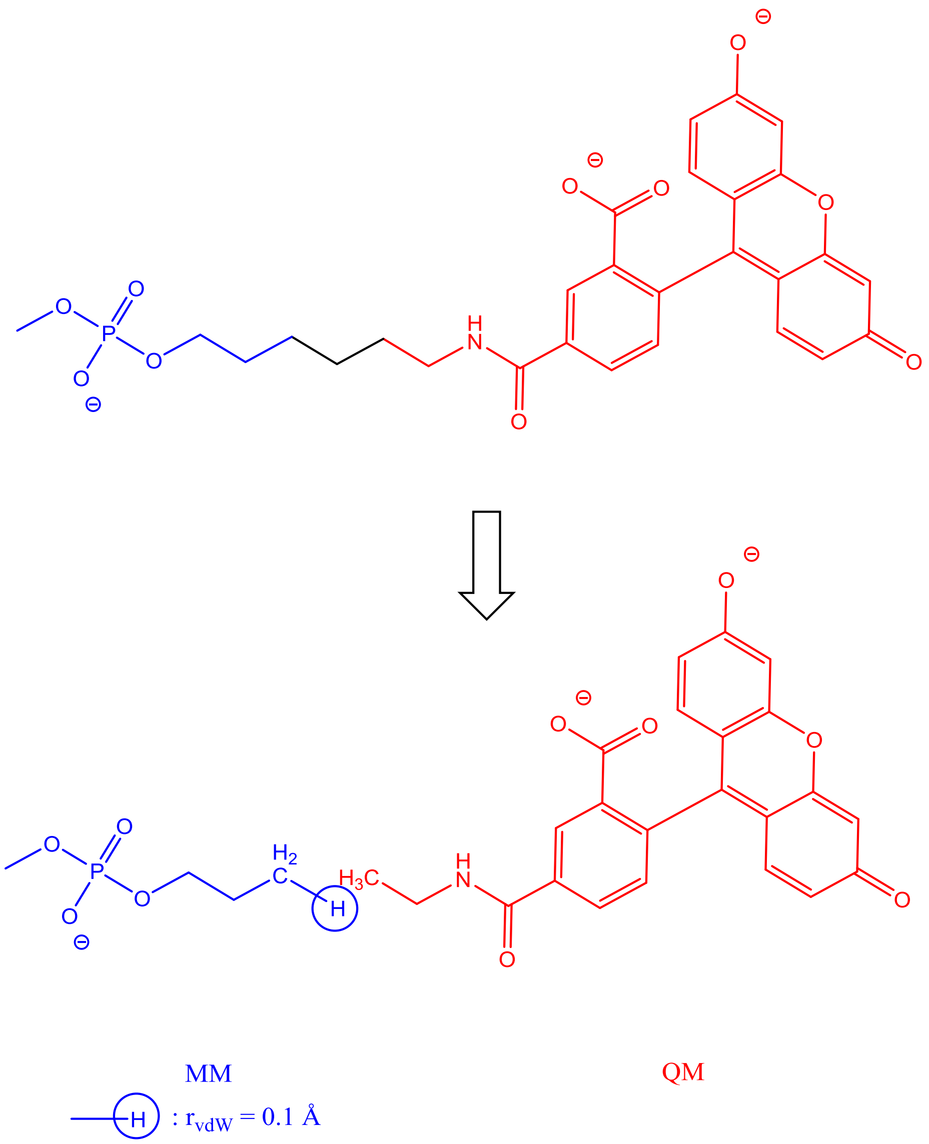

Fig. Y. System **1**, cutting scheme for QM/MM input preparation for the fluorescein chromophore. For more details on the QM/MM implementation, see reference [2].

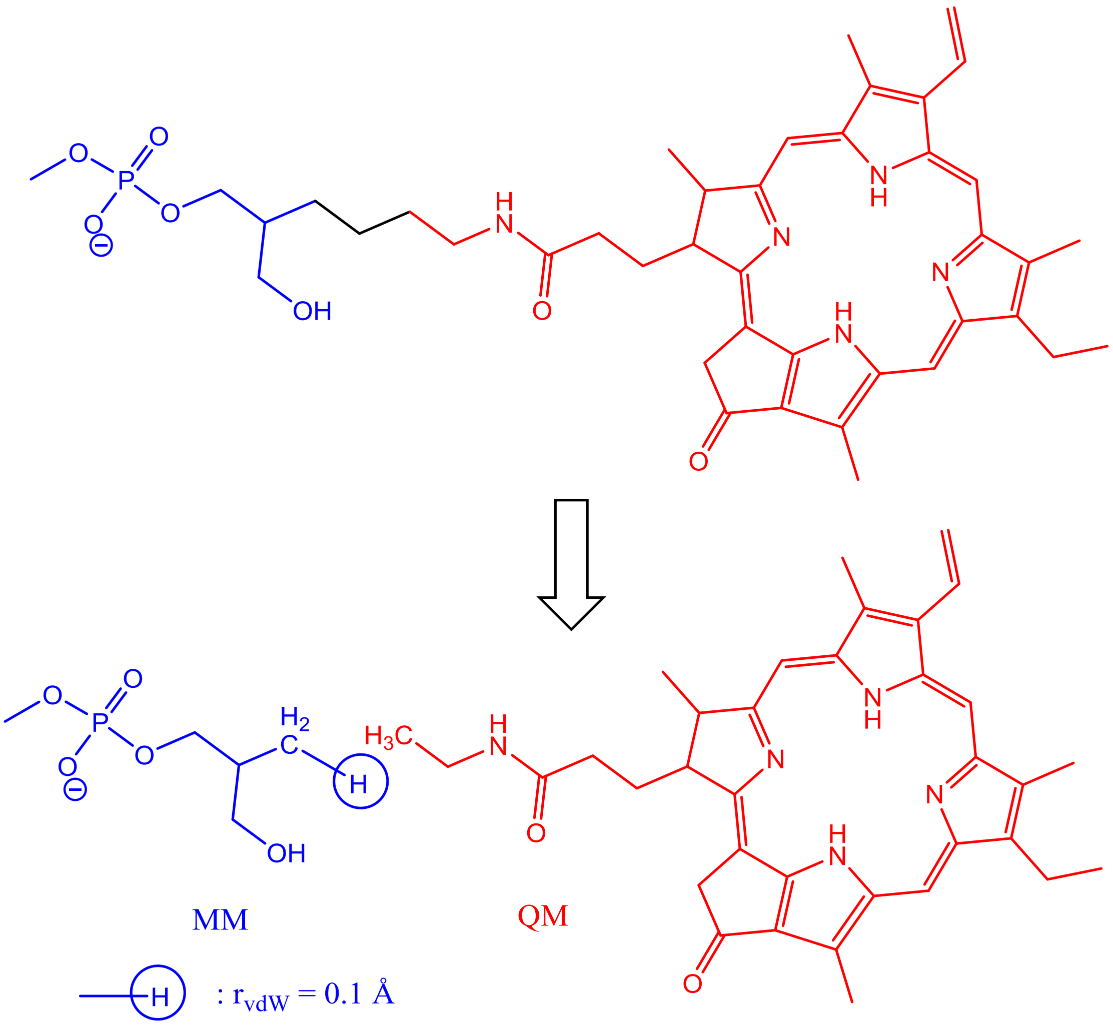

Fig. Z. System **1**, cutting scheme for QM/MM input preparation for the PPa chromophore. For more details on the QM/MM implementation, see reference [2].

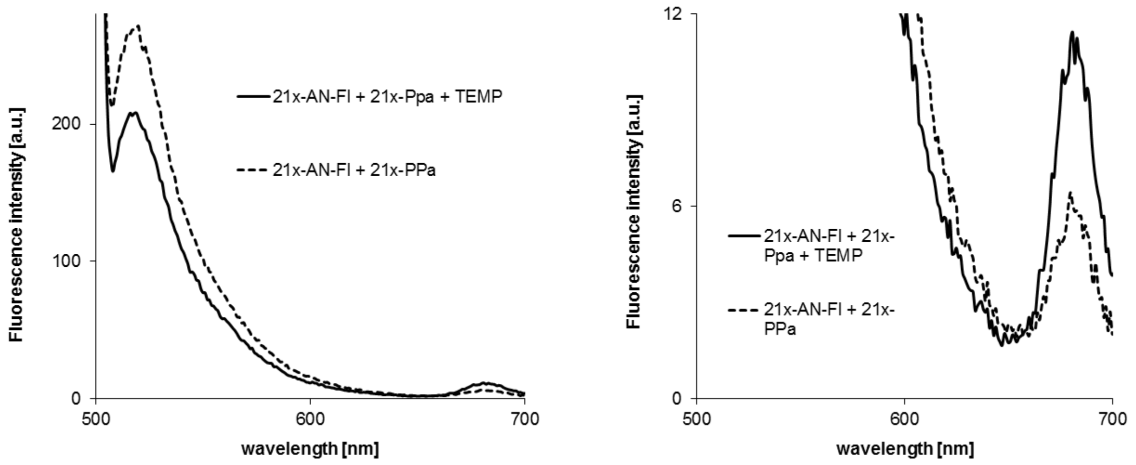

Fig. AA. Experimental evidence for FRET from fluorescein (Fl) to PPa in system **1**: Fluorescence spectrum showing donor and acceptor peaks before and after addition of template (TEMP, complementary DNA strand).

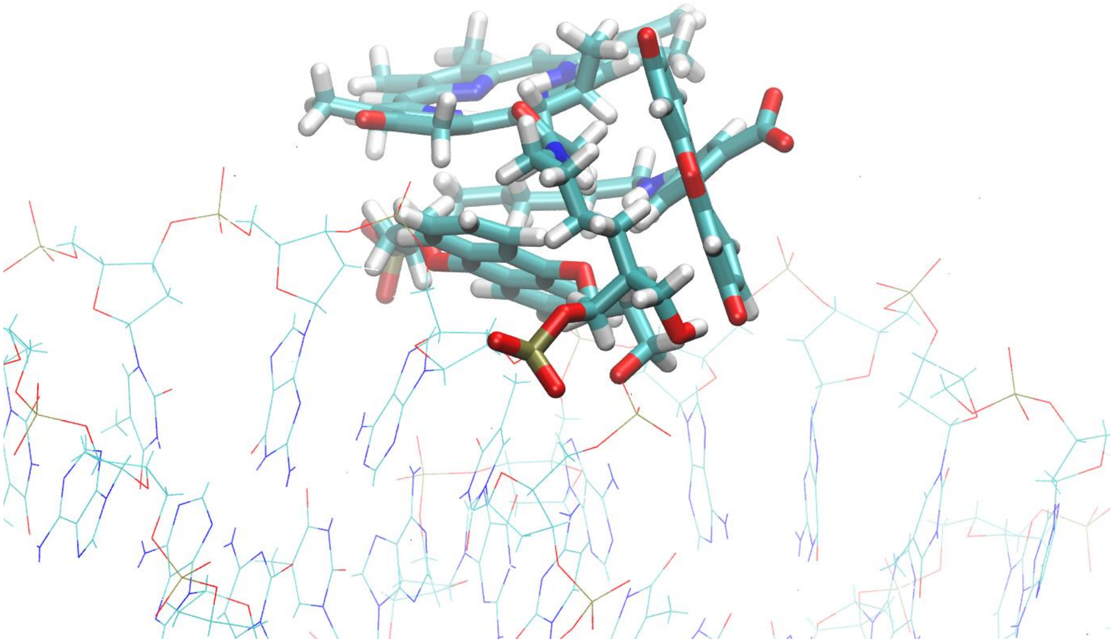

Fig. BB. MD simulation of system **1**, cluster analysis (RMSD-based clustering according to RMSD of PPa, anthracene and fluorescein ring heavy atoms, calculated vs. the minimized starting structure, after fitting the whole trajectory on the DNA backbone atoms of the minimized starting structure,[3] see main text for details): representative snapshot of cluster 0.

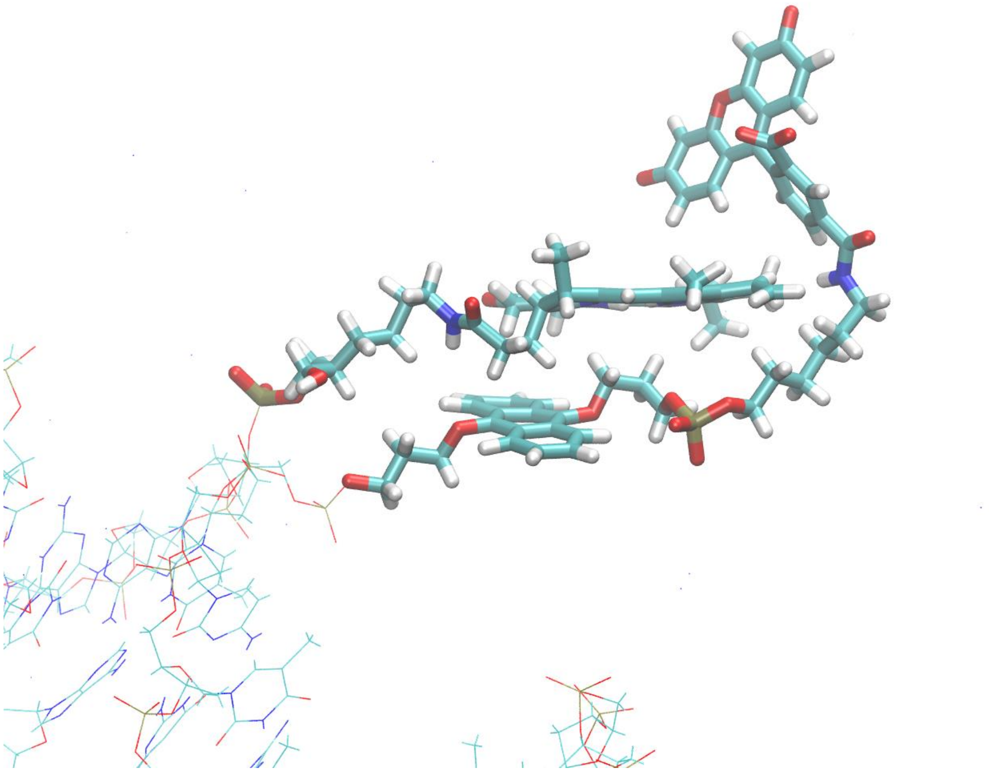

Fig. CC. MD simulation of system **1**, cluster analysis (RMSD-based clustering according to RMSD of PPa, anthracene and fluorescein ring heavy atoms, calculated vs. the minimized starting structure, after fitting the whole trajectory on the DNA backbone atoms of the minimized starting structure,[3] see main text for details): representative snapshot of cluster 1.

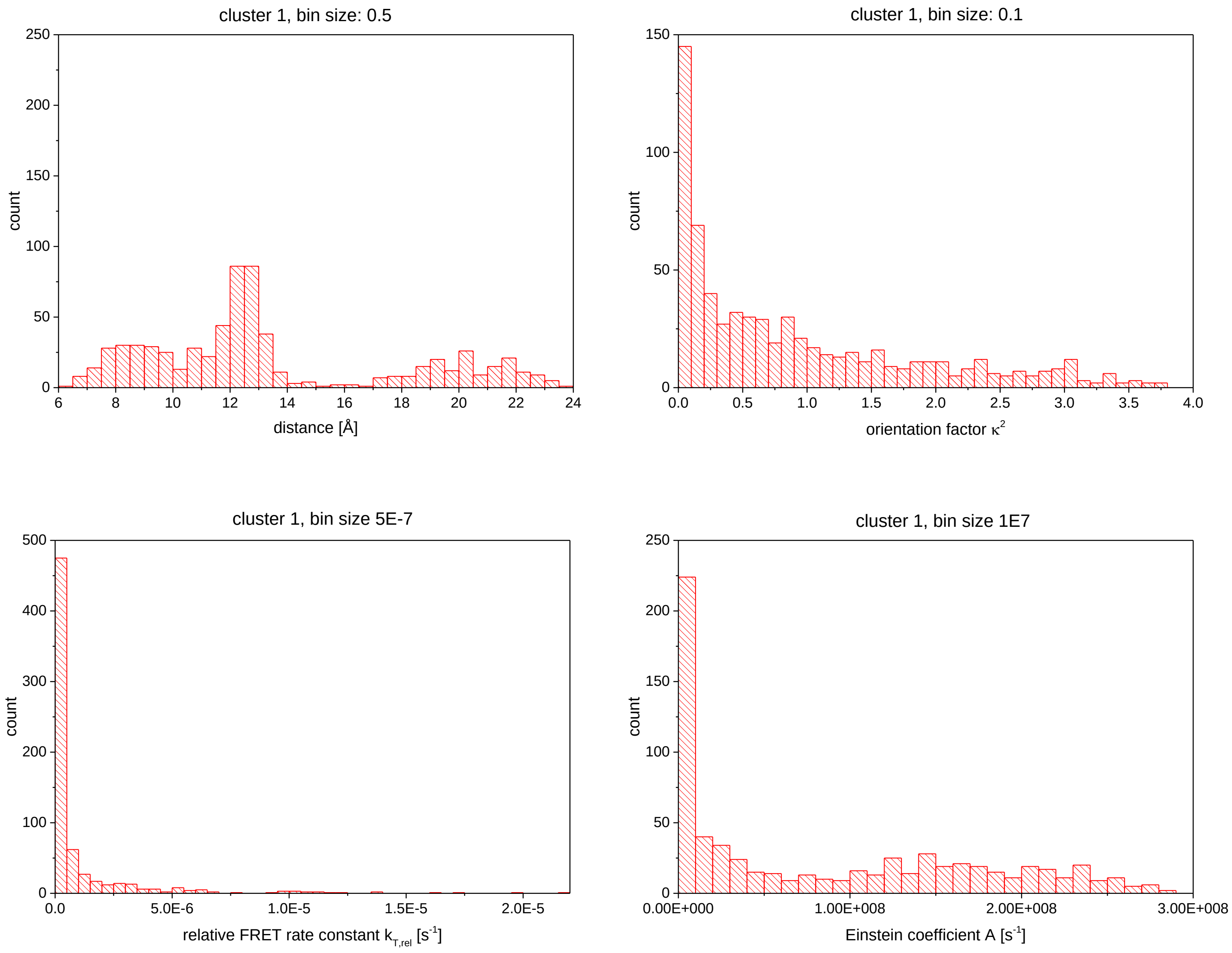

Fig. DD. QM/MM calculations of the snapshots of cluster 1 (MD simulation of system **1**): histograms of important quantities describing FRET.

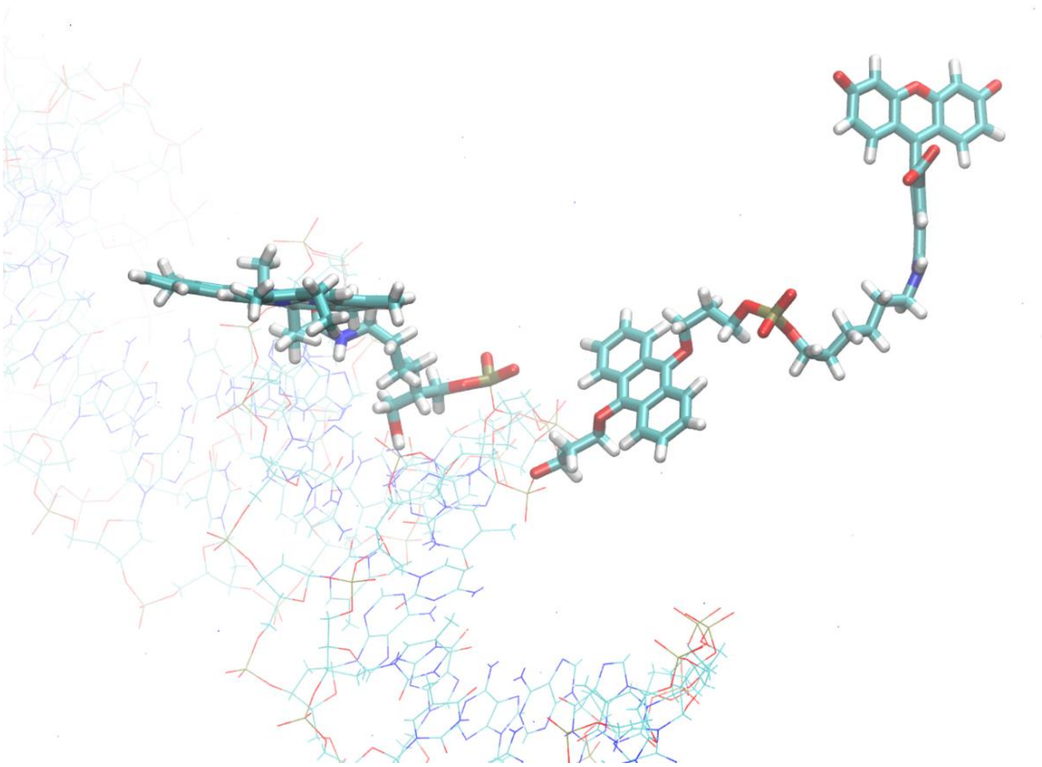

Fig. EE. MD simulation of system **1**, cluster analysis (RMSD-based clustering according to RMSD of PPa, anthracene and fluorescein ring heavy atoms, calculated vs. the minimized starting structure, after fitting the whole trajectory on the DNA backbone atoms of the minimized starting structure,[3] see main text for details): representative snapshot of cluster 2.

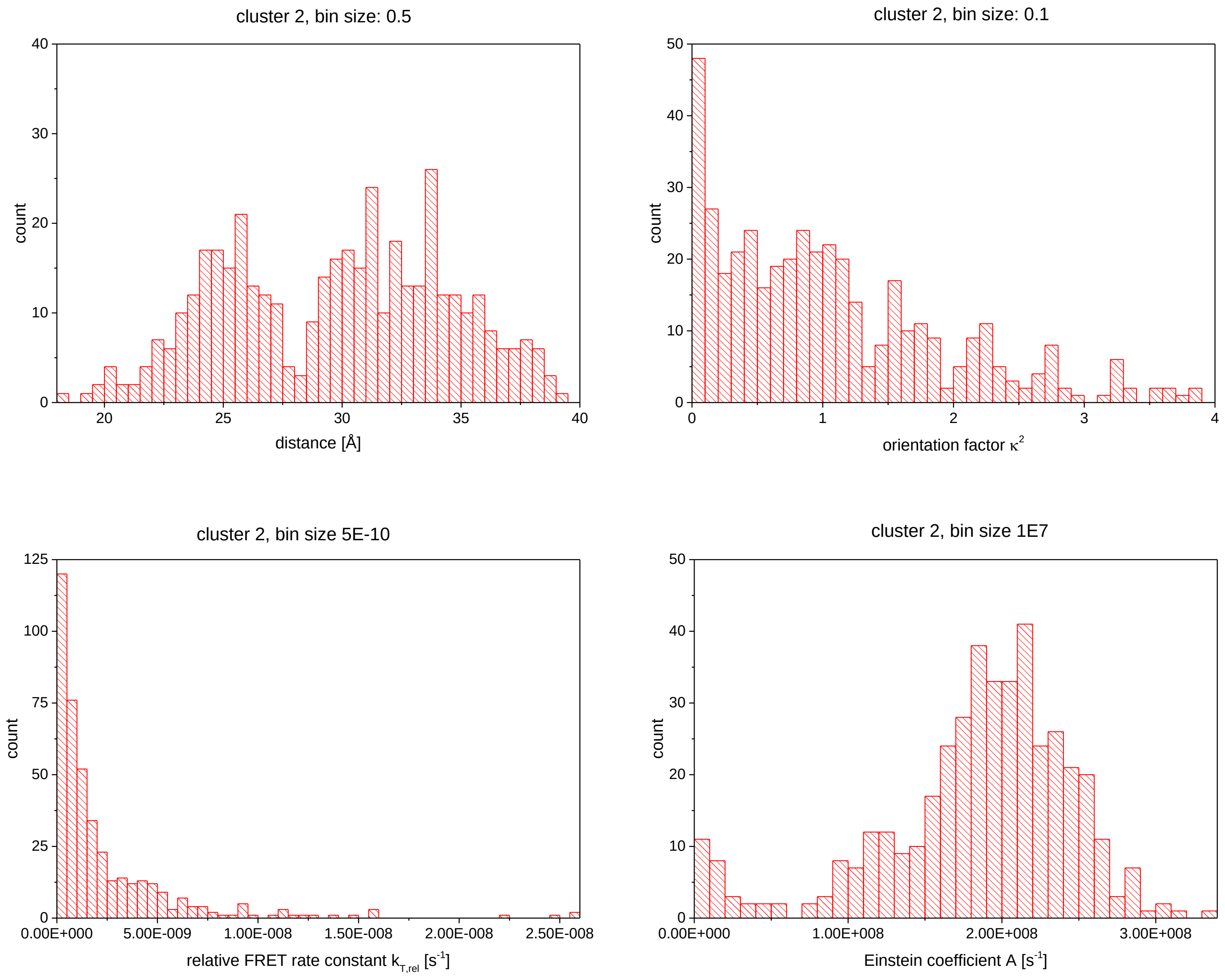

Fig. FF. QM/MM calculations of the snapshots of cluster 2 (MD simulation of system **1**): histograms of important quantities describing FRET.

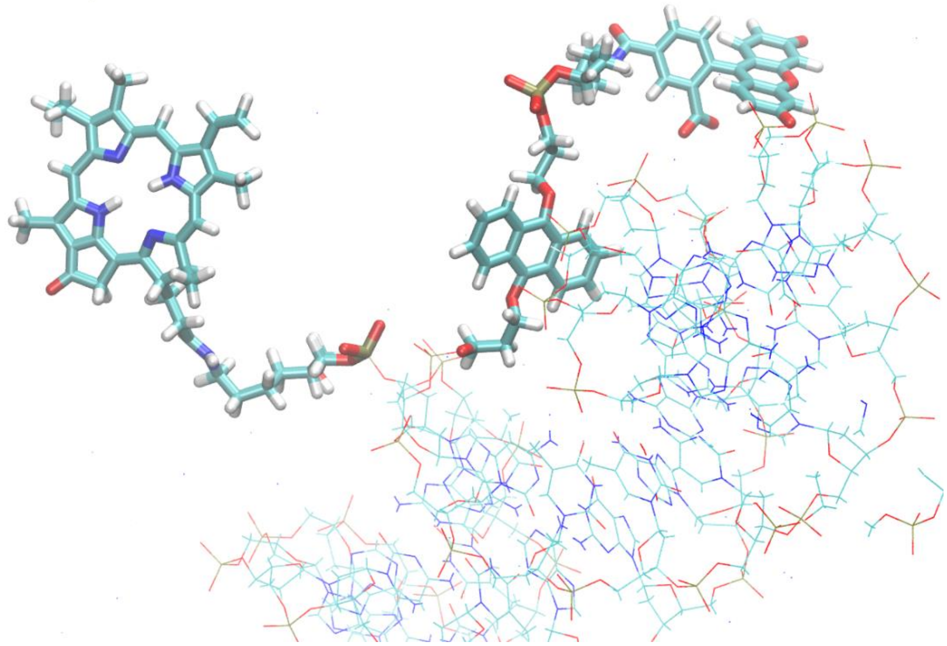

Fig. GG. MD simulation of system **1**, cluster analysis (RMSD-based clustering according to RMSD of PPa, anthracene and fluorescein ring heavy atoms, calculated vs. the minimized starting structure, after fitting the whole trajectory on the DNA backbone atoms of the minimized starting structure,[3] see main text for details): representative snapshot of cluster 3.

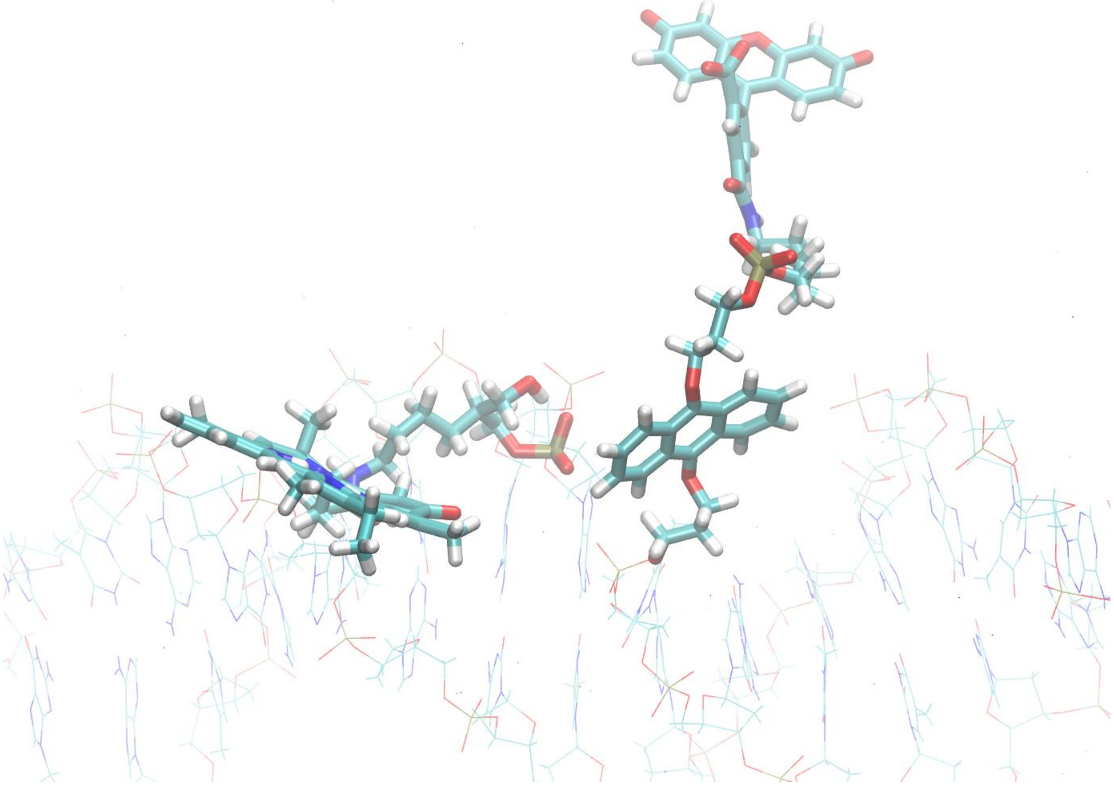

Fig. HH. MD simulation of system **1**, cluster analysis (RMSD-based clustering according to RMSD of PPa, anthracene and fluorescein ring heavy atoms, calculated vs. the minimized starting structure, after fitting the whole trajectory on the DNA backbone atoms of the minimized starting structure,[3] see main text for details): representative snapshot of cluster 4.

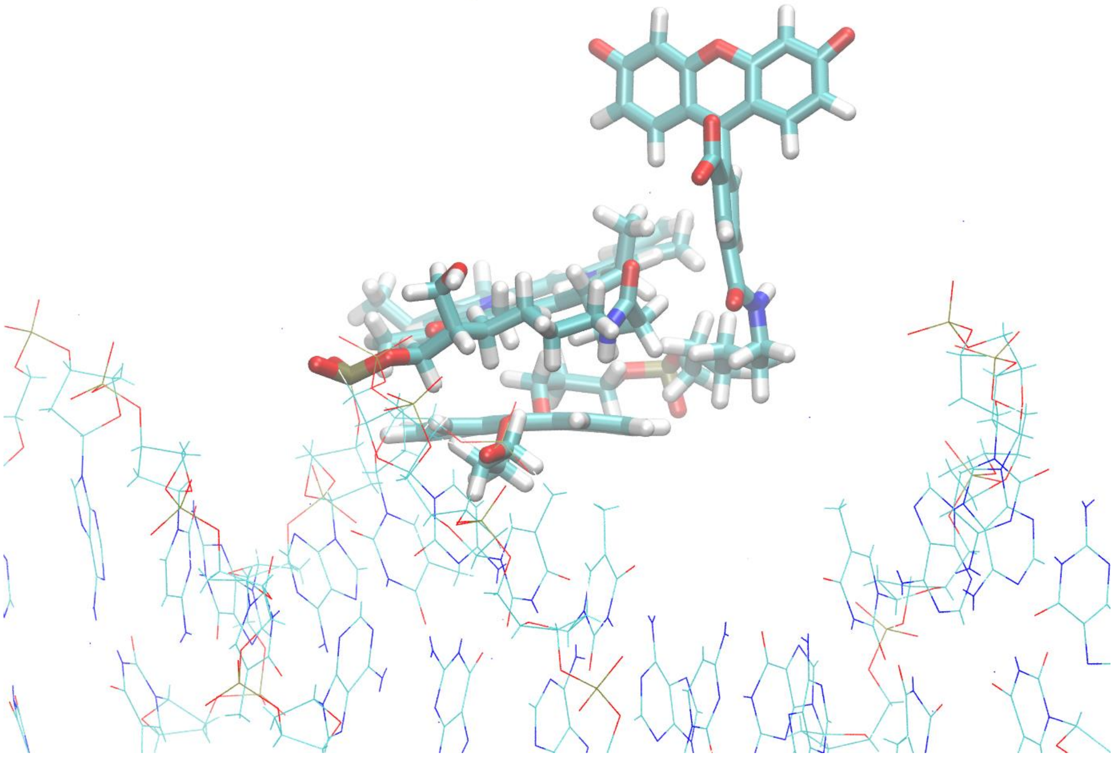

Fig. II. MD simulation of system **1**, cluster analysis (RMSD-based clustering according to RMSD of PPa, anthracene and fluorescein ring heavy atoms, calculated vs. the minimized starting structure, after fitting the whole trajectory on the DNA backbone atoms of the minimized starting structure,[3] see main text for details): representative snapshot of cluster 5.

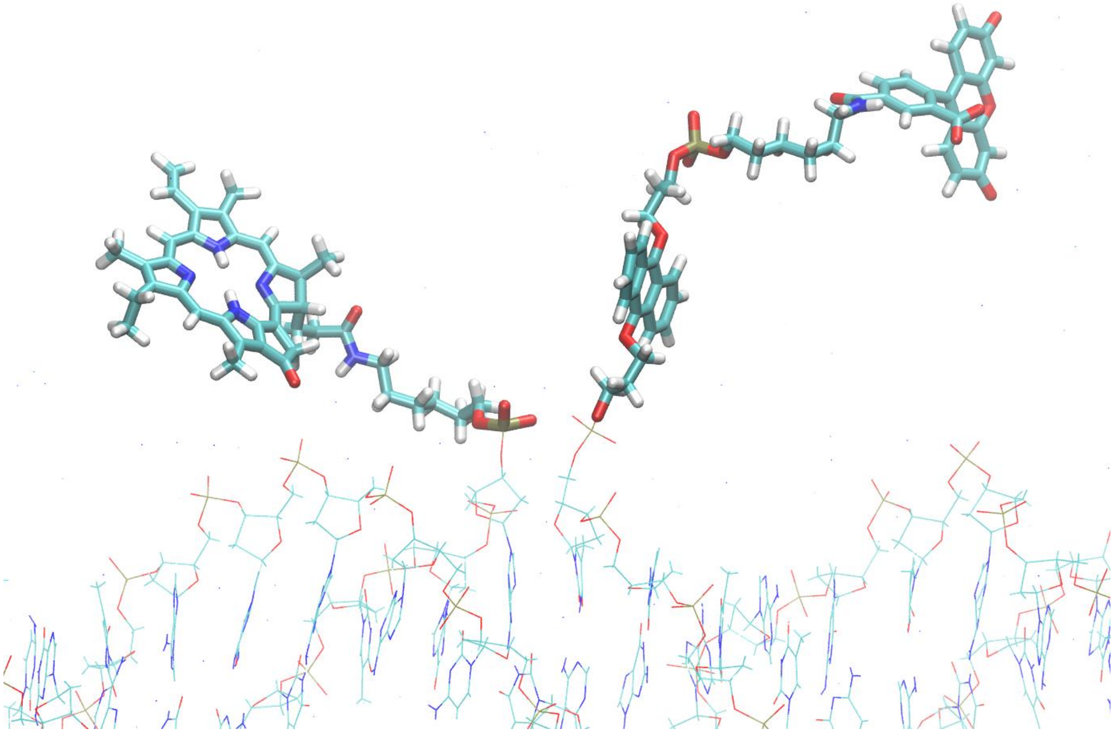

Fig. JJ. MD simulation of system **1**, cluster analysis (RMSD-based clustering according to RMSD of PPa, anthracene and fluorescein ring heavy atoms, calculated vs. the minimized starting structure, after fitting the whole trajectory on the DNA backbone atoms of the minimized starting structure,[3] see main text for details): representative snapshot of cluster 6.

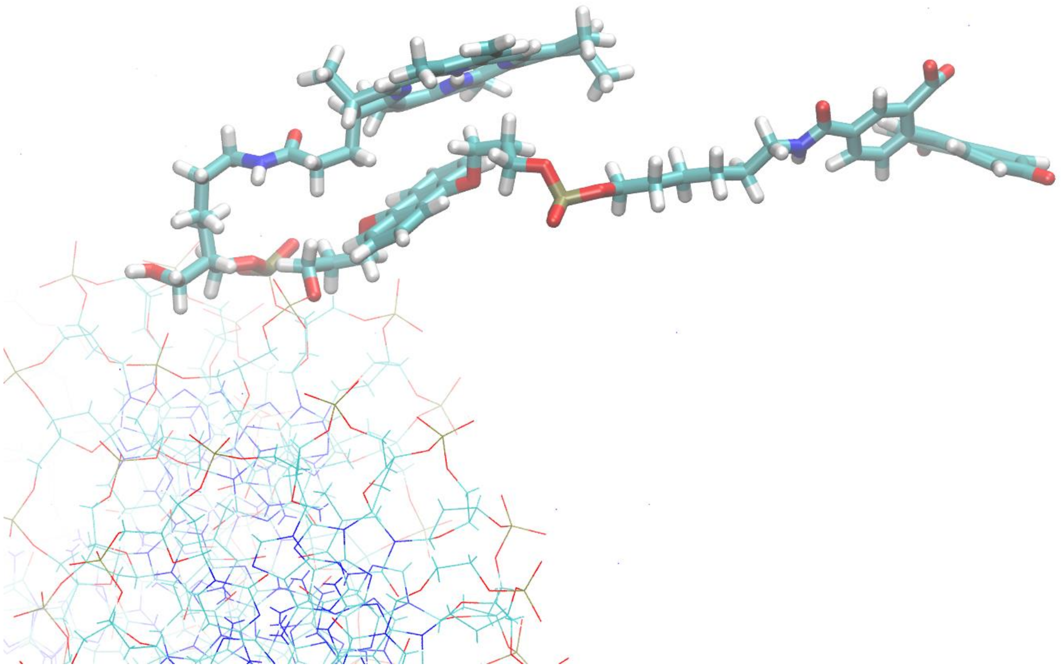

Fig. KK. MD simulation of system **1**, cluster analysis (RMSD-based clustering according to RMSD of PPa, anthracene and fluorescein ring heavy atoms, calculated vs. the minimized starting structure, after fitting the whole trajectory on the DNA backbone atoms of the minimized starting structure,[3] see main text for details): representative snapshot of cluster 7.

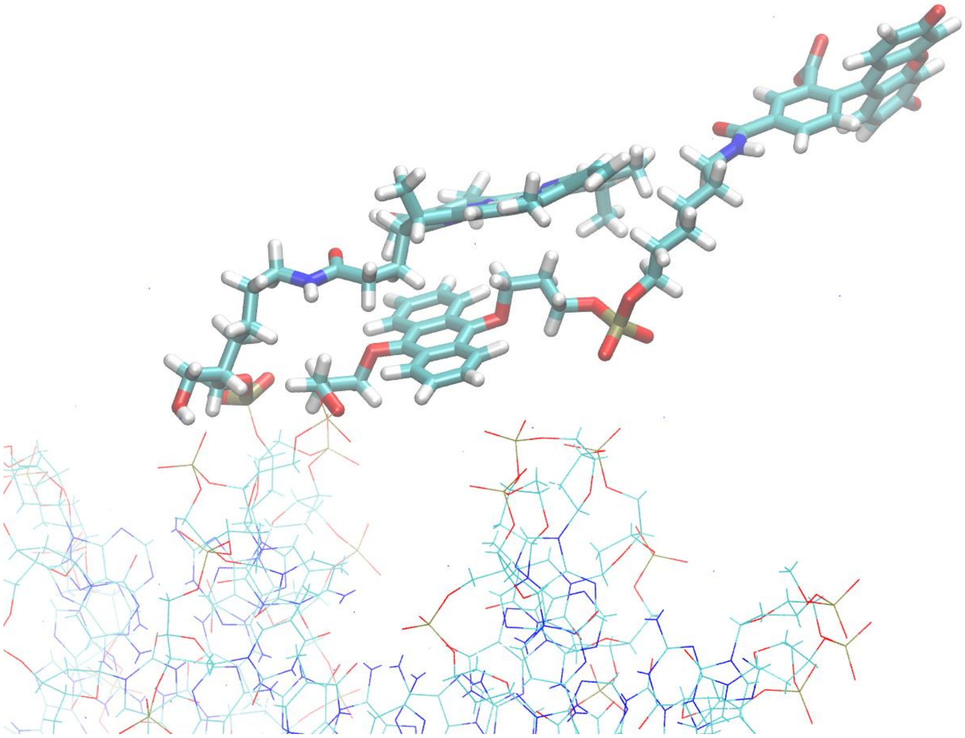

Fig. LL. MD simulation of system **1**, cluster analysis (RMSD-based clustering according to RMSD of PPa, anthracene and fluorescein ring heavy atoms, calculated vs. the minimized starting structure, after fitting the whole trajectory on the DNA backbone atoms of the minimized starting structure,[3] see main text for details): representative snapshot of cluster 8.

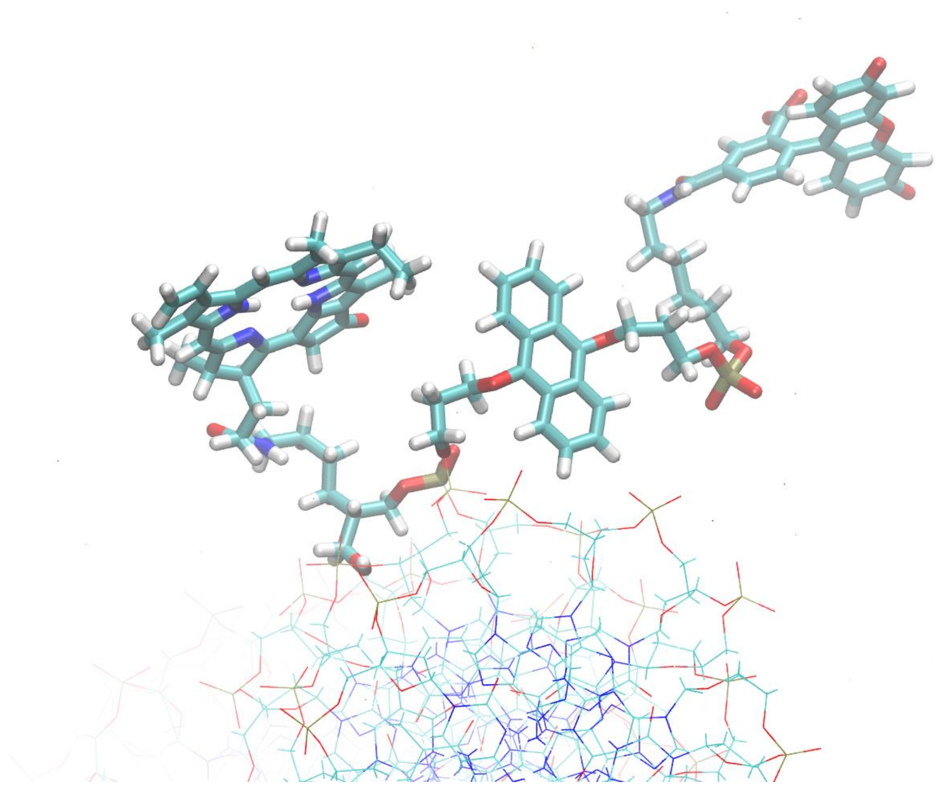

Fig. MM. MD simulation of system **1**, cluster analysis (RMSD-based clustering according to RMSD of PPa, anthracene and fluorescein ring heavy atoms, calculated vs. the minimized starting structure, after fitting the whole trajectory on the DNA backbone atoms of the minimized starting structure,[3] see main text for details): representative snapshot of cluster 9.

Nucleic Acid Force Fields

Although progress is being made,[4] MD simulations of nucleic acids, especially RNA, with classical force fields are not as reliable as protein simulations, which have now reached a very high level.[5, 6] One important reason for this is that the fixed, atom-centered point charges used in most current pair-additive force fields fail to describe the complex properties of the backbone accurately.[4] The force-field parameters for DNA and RNA are being refined constantly, and while DNA simulations can be performed with reasonable accuracy, for RNA the situation is more difficult. The Barcelona extension (α/γ dihedral modifications) to the Amber[7] ff94/ff99[8, 9] by Orozco and co-workers (ff99-bsc0) [10] has been tested intensively and shown to be state-of-the-art for simulating canonical DNA.[4] Recently, additional dihedral corrections have been added to the ff99-bsc0 force field, e.g. the ε/ζOL1[11] and χOL4[12] modifications for DNA. These are currently being tested intensively by several research groups, and will presumably become standard for most forms of DNA. ff99-bsc0 with χOL3 modifications is probably the best force field currently available for RNA.[13] However, none of these force fields is perfect and nucleic-acid force fields are subject to ongoing refinement.

For organic molecules, like the organic dyes investigated in this work, which are not parameterized in the standard force fields for proteins and nucleic acids, the general Amber force field (GAFF)[14] has proven to be very useful, maintaining compatibility with the biomolecular Amber force fields.

REFERENCES

1. Humphrey W, Dalke A, Schulten K. VMD - Visual Molecular Dynamics. J Molec Graphics. 1996;14:33-8.
2. Beierlein FR, Othersen OG, Lanig H, Schneider S, Clark T. Simulating FRET from Tryptophan: Is the Rotamer Model Correct? J Am Chem Soc. 2006;128(15):5142-52. doi: 10.1021/ja058414l.
3. Roe DR, Cheatham TE. PTRAJ and CPPTRAJ: Software for Processing and Analysis of Molecular Dynamics Trajectory Data. J Chem Theory Comput. 2013;9(7):3084-95. doi: 10.1021/ct400341p.
4. Šponer J, Banáš P, Jurečka P, Zgarbová M, Kührová P, Havrila M, et al. Molecular Dynamics Simulations of Nucleic Acids. From Tetranucleotides to the Ribosome. J Phys Chem Lett. 2014;5(10):1771-82. doi: 10.1021/jz500557y.
5. Lindorff-Larsen K, Maragakis P, Piana S, Eastwood MP, Dror RO, Shaw DE. Systematic Validation of Protein Force Fields against Experimental Data. PLoS ONE. 2012;7(2):e32131. doi: 10.1371/journal.pone.0032131.
6. Maier JA, Martinez C, Kasavajhala K, Wickstrom L, Hauser KE, Simmerling C. ff14SB: Improving the Accuracy of Protein Side Chain and Backbone Parameters from ff99SB. J Chem Theory Comput. 2015;11(8):3696-713. doi: 10.1021/acs.jctc.5b00255.
7. Case DA, Babin V, Berryman JT, Betz RM, Cai Q, Cerutti DS, et al. AMBER 14. University of California, San Francisco; 2014.
8. Cornell WD, Cieplak P, Bayly CI, Gould IR, Merz Jr. KM, Ferguson DM, et al. A second generation force field for the simulation of proteins and nucleic acids. J Am Chem Soc. 1995;117:5179-97.
9. Wang J, Cieplak P, Kollman PA. How Well Does a Restrained Electrostatic Potential (RESP) Model Perform in Calculating Conformational Energies of Organic and Biological Molecules? J Comput Chem. 2000;21(12):1049-74.
10. Pérez A, Marchán I, Svozil D, Sponer J, Cheatham III TE, Laughton CA, et al. Refinement of the AMBER Force Field for Nucleic Acids: Improving the Description of α/γ Conformers. Biophys J. 2007;92(11):3817-29.
11. Zgarbová M, Luque FJ, Šponer J, Cheatham TE, Otyepka M, Jurečka P. Toward Improved Description of DNA Backbone: Revisiting Epsilon and Zeta Torsion Force Field Parameters. J Chem Theory Comput. 2013;9(5):2339-54. doi: 10.1021/ct400154j.
12. Krepl M, Zgarbová M, Stadlbauer P, Otyepka M, Banáš P, Koča J, et al. Reference Simulations of Noncanonical Nucleic Acids with Different χ Variants of the AMBER Force Field: Quadruplex DNA, Quadruplex RNA, and Z-DNA. J Chem Theory Comput. 2012;8(7):2506-20. doi: 10.1021/ct300275s.
13. Zgarbová M, Otyepka M, Šponer J, Mládek A, Banáš P, Cheatham TE, et al. Refinement of the Cornell et al. Nucleic Acids Force Field Based on Reference Quantum Chemical Calculations of Glycosidic Torsion Profiles. J Chem Theory Comput. 2011;7(9):2886-902. doi: 10.1021/ct200162x.
14. Wang J, Wolf RM, Caldwell JW, Kollman PA, Case DA. Development and Testing of a General Amber Force Field. J Comput Chem. 2004;25:1157-74.
